# Zinc-Mediated Defenses Against Toxic Heavy Metals and Metalloids: Mechanisms, Immunomodulation, and Therapeutic Relevance

**DOI:** 10.3390/ijms26199797

**Published:** 2025-10-08

**Authors:** Roopkumar Sangubotla, Shameer Syed, Anthati Mastan, Buddolla Anantha Lakshmi, Jongsung Kim

**Affiliations:** 1 Department of Chemical, Biological, and Battery Engineering, Gachon University, 1342, Seongnam Daero, Seongnam-si 13120, Gyeonggi-Do, Republic of Korea; gachonroop@gmail.com; 2 Key Laboratory of Ecological Safety and Sustainable Development in Arid Lands, Northwest Institute of Eco-Environment and Resources, Chinese Academy of Sciences, Lanzhou 730000, China; 3 Drylands Salinization Research Station, Northwest Institute of Eco-Environment and Resources, Chinese Academy of Sciences, Lanzhou 730000, China; 4 Department of Microbiology, School of of Sciences, Jain (Deemed to be University), Bengaluru 560027, Karnataka, India; mastan.anthati@gmail.com; 5 Department of Electronic Engineering, Gachon University, 1342 Seongnam-Daero, Seongnam-si 13120, Gyeonggi-Do, Republic of Korea; mamathab72@gmail.com; 6 Department of Semiconductor Engineering, Gachon University, 1342 Seongnam-Daero, Seongnam-si 13120, Gyeonggi-Do, Republic of Korea

**Keywords:** heavy metal toxicity, zinc, protective role, metallothioneins, antioxidative, human health

## Abstract

Zinc (Zn), a naturally occurring trace element ubiquitous in the Earth’s crust, soil, and water, is indispensable for human health due to its physiological and nutritive benefits. In this scenario, Zn is pivotal for maintaining homeostasis against toxic effects exerted by heavy metals (HMs) through bioaccumulation and metabolic interference. Zinc is an enticing cofactor for miscellaneous biochemical enzymes such as Zn metalloenzymes, which mediate crucial cellular processes, including cell proliferation, protein synthesis, immune modulation, epigenetic regulation, and nucleic acid synthesis. Recently, several research studies have focused on the thorough investigation of Zn supplementation in controlling HM toxicity by competing for binding sites and boosting protective mechanisms in humans. The current article discusses the upper limits for various toxic HMs in staple crop foods, as provided by globally recognized organizations. Clinical studies recommend a daily dose of 11 mg of Zn for healthy men and 8–12 mg for women in healthy and pregnancy conditions. However, during Zn deficiency, therapeutic supplementation is expected to be adjustable, and the dosage is increased from 15 to 30 mg daily. This review discusses the dysregulation of specific Zn importers and transporters (ZIPs/ZnTs) due to their clinical significance in immune system dysfunction as well as the progression of a myriad of cancers, including prostate, breast, and pancreas. Moreover, this review emphasizes indispensable in vitro and in vivo studies, as well as key molecular mechanisms related to Zn supplementation for treating toxicities exacerbated by HMs.

## 1. Introduction

Over the past century, rapid industrialization and human activities have significantly increased the exploitation of natural resources, leading to environmental pollution and posing a severe threat to biodiversity and ecosystems [1]. Among the various pollutants, heavy metals (HMs) and metalloids such as cadmium (Cd), lead (Pb), chromium (Cr), mercury (Hg), and arsenic (As) are particularly concerning due to their environmental persistence and possible toxicity at higher concentrations. Heavy metals and metalloids can damage cells through robust interaction with biomolecules, metal displacement, and oxidative stress, ultimately accumulating in terrestrial ecosystems primarily as a result of anthropogenic activities [2]. The excessive release of HMs/metalloids mainly originates from food and water contamination and polluted air generated by mining, smelting, pharmaceutical, and manufacturing industries, as well as from the use of fertilizers and pesticides in agriculture [3,4,5,6]. The excessive contamination by HMs/metalloids such as Cd, Pb, and As can adversely affect human health. These HMs/metalloids are intrusively common in staple crops like rice and wheat, which serve as a major source of dietary exposure [7,8,9,10]. In addition, previous studies also demonstrated that the excessive concentrations of HMs/metalloids in the diet lead to health-related complications, and thus, it is critical to regulate their levels in the diet [11,12,13,14,15]. In this context, the Food Safety and Standards Authority of India (FSSAI) establishes maximum permitted limits for Cd at 0.4 mg/kg in rice and Pb at 0.2 mg/kg in cereal grains [16]. China specifies the maximum permitted limits for Cd and inorganic Pb at 0.2 mg/kg for rice and 0.2 mg/kg for cereals [17,18]. While European nations set permitted thresholds for inorganic As at 0.15 mg/kg in polished rice [19,20,21]. The Food and Drug Administration (FDA) in the United States has set allowable levels of 20 ppb for Pb in dry infant cereals [22,23]. Therefore, a proper regulation of contaminated levels of HMs/metalloids in the consumed diet can ensure human health.

Based on the above discussion, it is necessary to understand the significance of Zn supplementation in alleviating HMs/metalloid toxicity. For instance, under extreme stress conditions, ZnO NPs have demonstrated decreased levels of oxidative stress markers like H_2_O_2_ and malondialdehyde (MDA) in black gram seedlings and alleviated As-stress. Furthermore, the concentration-dependent ZnO NPs (300 mg/L) have mitigated As-induced toxicity in *Pisum sativum* by inhibiting the arsenic absorption, lessening oxidative stress, genotoxicity, and programmed cell death [24,25]. In this regard, the application of ZnO NPs has been investigated as one of the possible strategies to alleviate stress from HMs/metalloids in crops and humans, mostly by competing for transporters, inducing metallothioneins (MTs), and enhancing antioxidant defenses. Nonetheless, their efficacy is not universal and is contingent upon crop species, ambient conditions, dosage, and a person’s health status. More importantly, the clinical safety of ZnO NPs is still under active investigation, and thus their application needs to be considered experimental rather than established.

**Figure 1:**
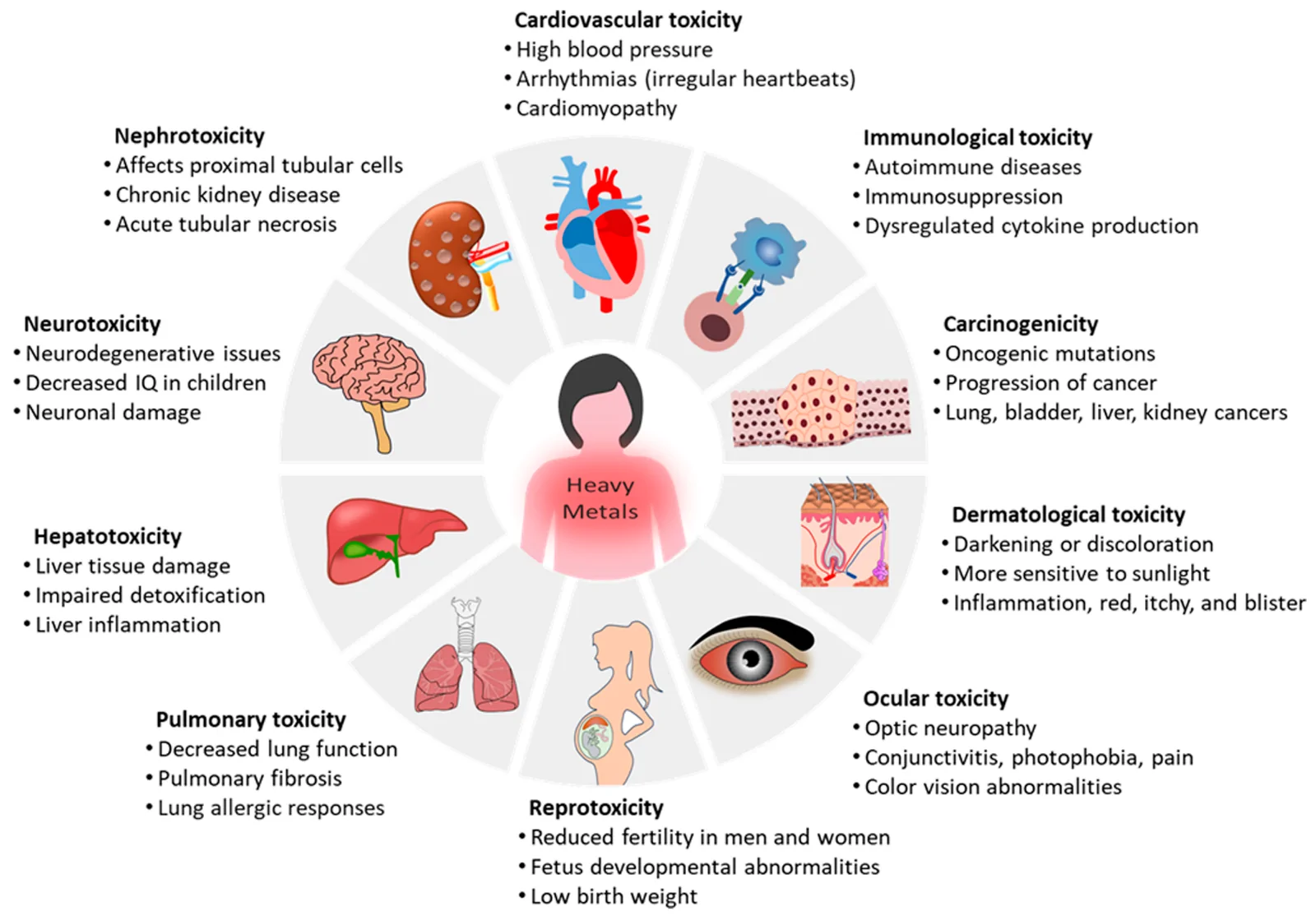
Complications associated with heavy metal exposure, including cancer, neurological disorders, GI diseases, kidney problems, skin lesions, vascular damage, and immune system dysfunction.

The toxicity from HMs/metalloids is primarily linked to significant health hazards due to their interference in biological functions, posing a serious threat to human health. For instance, HMs interfere with normal biological mechanisms and mimic essential elements, subsequently disrupting metabolic functions [26]. Arsenic, Cd, Pb, methylmercury, and hexavalent chromium [Cr(VI)] are among the most significant toxic metals/metalloids associated with human poisoning. Their toxicokinetic features signify the fate of poisoning and depend on the type of excretion: for example, some HMs, such as copper, methylmercury, and manganese, are effectively excreted through biliary excretion, while others (e.g., Pb, As, Cr(VI), and Cd) are excreted through urinary excretion. More importantly, their long-term exposures have been correlated with gastrointestinal and renal dysfunction, cardiovascular damage, immunological dysregulation, cancer, neurotoxicity, and congenital abnormalities [27,28] (Figure 1). Depending on their concentrations, minimal to moderate exposure to HMs alone or in combination can exacerbate these health issues [29,30,31,32]. In children, chronic low-dose exposure poses a subtle yet significant risk, leading to neuropsychiatric disorders, reduced intelligence quotient (IQ), fatigue, and anxiety [33,34]. Moreover, certain HMs are recognized as human carcinogens, producing reactive oxygen species (ROS), which impair host defense mechanisms, causing genomic instability, and can lead to cancer [35].

By accounting for significant health risks, it is crucial to explore protective strategies against HM toxicity. Zinc, an essential trace element, is exploited in multifaceted pathological processes: cell function, immune development, and the synthesis of proteins and nucleic acids. Zinc can mitigate the toxic effects of HMs in humans by competing with them for binding sites and enhancing metallothionein (MT) and antioxidant activities. Thus, adequate Zn intake through diet or supplements may be a vital strategy for mitigating the harmful effects of HM exposure. More importantly, Zn deficiency is a recognized nutritional problem worldwide, affecting both developed and developing countries. Since Zn was first identified as a crucial micronutrient for humans in 1961, its deficiency has been associated with miscellaneous symptoms [36,37]. These include skin lesions and diarrhea, and specifically, severe manifestations such as anemia, growth retardation, mental lethargy, hyperzincuria, infertility, hyperammonemia, and thymic atrophy.

In this review, we emphasized the value of Zn supplementation against HM toxicity for improving health benefits. We discussed the significance of Zn deficiency and its adverse effects on human health and thoroughly explained the multifarious biological potentials of Zn in apoptosis, the immune system, neuronal death, cardiovascular diseases, cancer, and aging. Moreover, this review focuses on the key molecular mechanisms by which Zn affects immune functions, as well as the physiological roles of Zn importers (ZIPs) and transporters (ZnTs) in maintaining Zn homeostasis and addressing Zn deficiency disorders. The deficiency of ZIP1 in promoting prostate cancer and the therapeutic roles of ZIP6, ZIP7, and ZIP10 in breast cancer, as well as the overexpression of ZIP4 promoting pancreatic cancers, were thoroughly discussed. Evidently, typical molecular mechanisms, in vitro, and in vivo studies related to the Zn supplementation against HM toxicity were reviewed.

## 2. Suppressive Effects of Heavy Metals on the Immune System

Metals are ubiquitous in the environment, present in the atmosphere, the Earth’s crust, and water bodies, and accumulate in various biological organisms, including micro- and macroorganisms. Heavy metals, defined as those with a density above 5 g/cm^3^ and an atomic weight exceeding 40.04, include some HMs that are essential for physiological and biochemical functions, which exert adverse effects on ecosystems and living organisms, contributing to mental disorders, blood damage, and organ impairment, including the kidney and liver, leading to various diseases, including neurodegenerative (ND) conditions [26,38,39].

**Figure 2:**
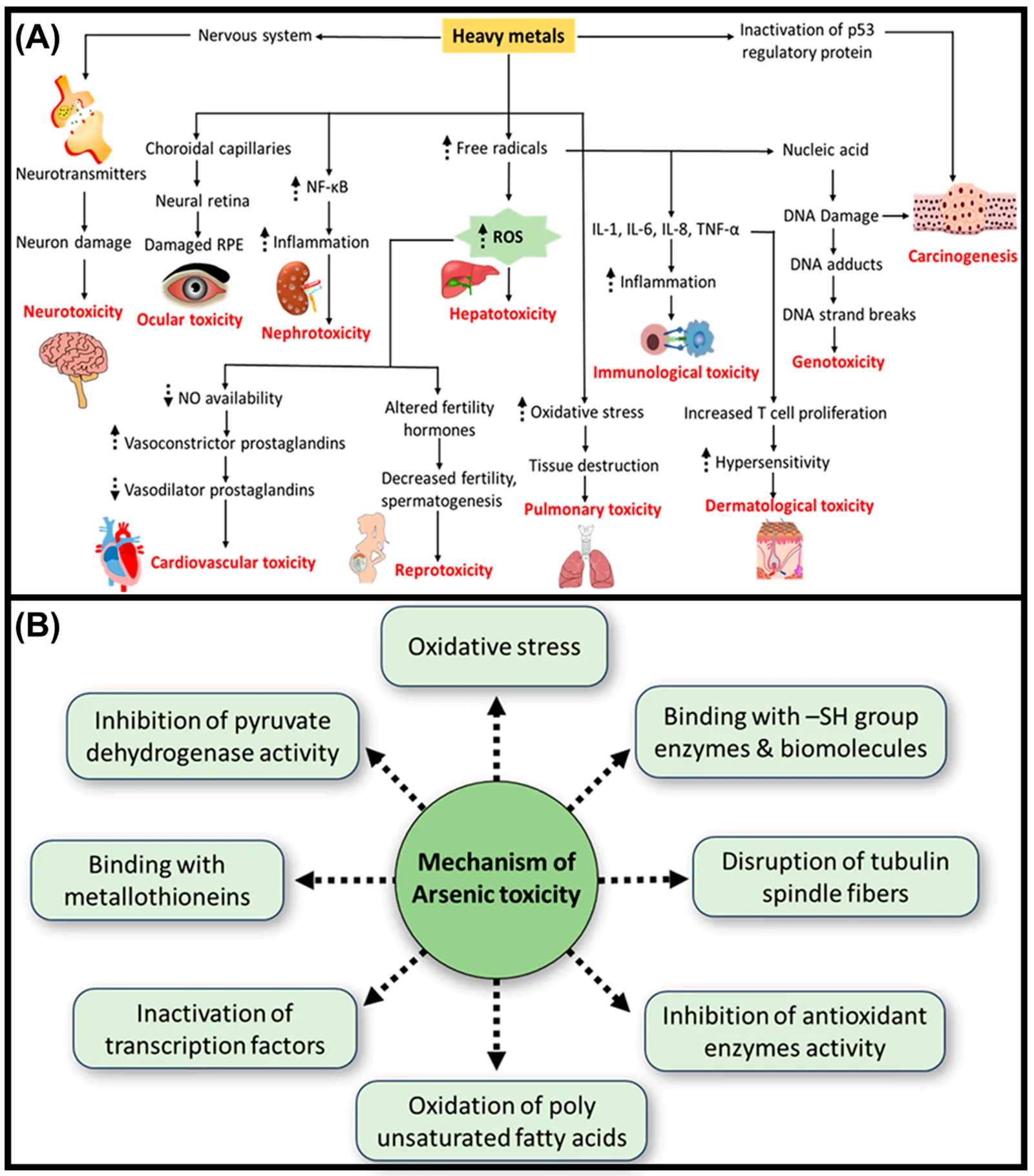
(**A**) Mechanistic overview of key pathogenic responses to heavy metal toxicity. (**B**) Schematic illustration depicting different mechanisms for arsenic toxicity in the human body.

Heavy metal toxicity primarily manifests through the generation of ROS, oxidative stress, and consequent adverse health effects. Accumulation of HMs, such as Pb, As, Hg, Cd, Cr, and Ni, can disrupt the body’s essential metabolic processes. Carcinogenic metal ions, particularly Ni and As, trigger redox reactions in biological systems, generating free radicals (FRs) that oxidize proteins and DNA. As illustrated in Figure 2A, the accumulation of HMs leads to the generation of ROS, contributing to oxidative stress and the onset of various diseases.

Arsenic exhibits semi-metallic properties and is notably toxic and carcinogenic, with its counterparts, including arsenite (As^3+^) and arsenate (As^5+^), being particularly harmful for human lives. Humans can ingest As through industrial sources, accidental exposure, or the use of arsenical insecticides, which can contaminate drinking water. Acute poisoning can occur through intentional ingestion during suicide attempts or accidental ingestion by children. Its exposure has detrimental effects on cells by targeting sulfhydryl (R-SH) moieties, impairing fundamental metabolic activities [40,41,42,43]. Both inorganic arsenic species, including arsenite As (III) and arsenate As (V), undergo methylation through microorganisms and humans, resulting in the production of monomethylarsonic acid (MMA) and dimethylarsinic acid (DMA). During bioconversion, As^2+^ ions are enzymatically transformed to methylated arsenic forms; those serve as biomarkers of chronic As^2+^ exposure. Methylated inorganic arsenic, excreted in the urine as MMA(V) and DMA(V), indicates chronic As^2+^ exposure; however, the intermediate product [MMA(III)] is not eliminated and remains within cells. Among these compounds, MMA (III) is more toxic than other arsenicals and may be the primary chemical cause of arsenic-induced cancer [41]. A myriad of toxicity mechanisms, including oxidative stress, DNA damage, epigenetic modifications, mitochondrial dysfunction, altered signal transduction pathways, and impaired cellular metabolism, are depicted in Figure 2B.

The Joint Expert Committee on Food Additives and Contaminants (JECFA) from the Food and Agriculture Organization (FAO) and World Health Organization (WHO) established a tolerable intake level of Cd at 7 µg/kg body weight/week, a monthly intake of Cd at 25 µg/kg body weight, and 0.83 µg/kg body weight/day [44]. From the toxicokinetic data, the cadmium excretion threshold of 5.24 μg/g creatinine was allowed for nephrotoxicity [45]. While, for methylmercury, the intake levels are as follows: 1.6 µg/kg body weight/week, 6.9 µg/kg body weight/month, and 0.23 µg/kg body weight/day. For inorganic mercury (Hg^2+^) species, the intake levels are 4.0 µg/kg body weight/week, 17 µg/kg body weight/month, and 0.57 µg/kg body weight/day. However, for Pb and As, there are no specified values given by JECFA on this basis [46].

**Figure 3:**
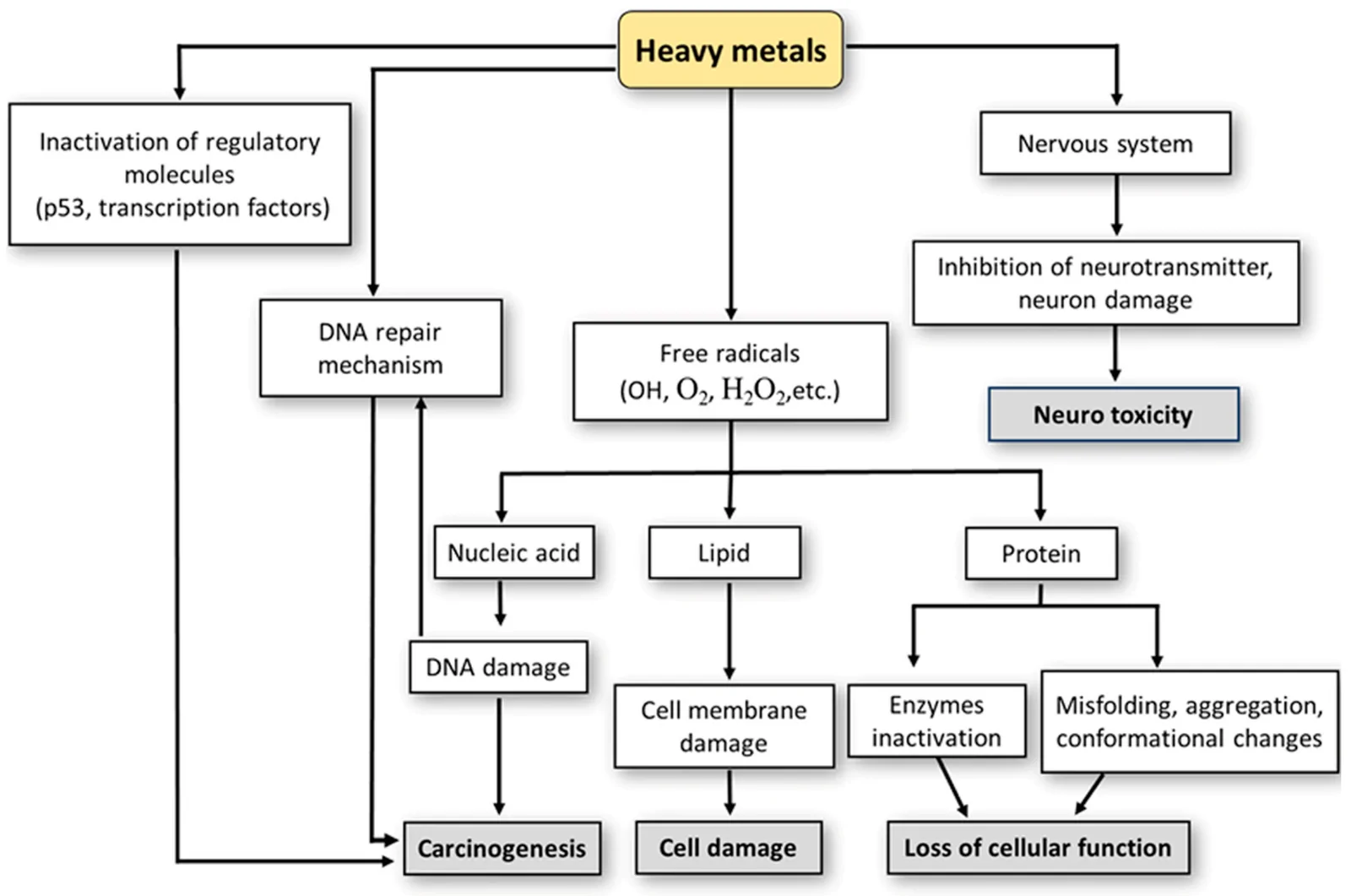
Effects of heavy metal exposure on human metabolism and the immune system. Reproduced under the terms of the Creative Commons Attribution 3.0 License. Adapted from [47].

Noteworthy, inflammation is a stringent physiological process that defends tissues against injury and infection and persistently produces associated cytokines and chemokines [26,38,39]. In this regard, HM exposure profoundly impacts the immune system, causing lowered immune cell counts and functional damage to various immune cells, resulting in detrimental health effects (Figure 3) [47]. More importantly, chronic exposure to HMs disrupts both innate and adaptive immune systems, typically inducing inflammatory reactions.

The innate immune system constitutes the body’s first line of defense against pathogens, involving various phagocytic cells such as basophilic and eosinophilic granulocytes, dendritic cells, neutrophils, macrophages, mast cells, and NK cells [48]. These cells cooperate with adaptive immune cells, including T and B lymphocytes, to initiate targeted immune responses against infections. The International Agency for Cancer Research (IACR) classifies Cd and its allied substances as group 1 carcinogens, whereas Pb is categorized as a “possible” human carcinogen (group 2A) [49]. Acute Cd exposure impacts the immune system and cellular structures by increasing the formation of ROS, hence inducing oxidative stress. Moreover, Cd induces apoptosis in T lymphocytes by harnessing oxidative stress and downregulating cytokines triggered by T cells. The biological half-life of Cd in blood and renal cortex is expected to range from 3 to 4 months and 10 to 30 years, respectively. Biologically, its exposure adversely affects the quantity, maturation, and functionality of T-cells via intricate effects on phagocytic and innate immune cells [50]. Furthermore, the half-life of Pb in the blood of adults is approximately 32 days. Acute Pb exposure can generate oxidative stress by promoting ROS and is capable of modifying the functionality of T cells [51]. Altogether, Cd and Pb negatively impact the immunological system by enhancing the expression of certain inflammatory mediators, altering immunological responses, lymphocyte activity, and cytokine production.

Phagocytosis, a crucial process in innate immunity, eliminates pathogens and cellular debris [52]. However, HM exposure has been shown to impair phagocytic activity. For instance, CdCl_2_ inhibits neutrophil phagocytic activity, while Pb exposure decreases phagocytic activity in human neutrophils [53]. Macrophages, key players in both innate and adaptive immunity, play a crucial role in homeostatic, immunological, and inflammatory processes. Inorganic Hg impairs macrophages’ ability to produce nitric oxide against bacteria, and variations in species sensitivity have been observed in alveolar macrophages of animals exposed to Ni-Cu metals [54]. Natural killer cells, cytotoxic large granular lymphocytes crucial in the defense against cancer cells, exhibit suppressed activity following exposure to Ni compounds [55]. This highlights the significance of understanding HM immunotoxicity and implementing measures to mitigate their adverse effects on physiological and immune function.

## 3. Importance of Zinc in Biological Functions

Zinc is a crucial micronutrient and an integral component of numerous transcription factors and proteins. Over 10% of eukaryotic cell proteins are bound by Zn ions, reflecting its essential role in controlling enzyme activity and regulating various metabolic processes across diverse cell types [39]. Zinc is indispensable for immunity, growth and development, neurological function, and reproduction. Zinc deficiency compromises both innate and adaptive immune systems, increasing susceptibility to infectious illnesses. Furthermore, Zn has anti-inflammatory and antioxidant characteristics essential for regulating immunological responses. Metallothioneins and Zn-binding proteins play a major role in cellular homeostasis and facilitate therapeutic protection against oxidative stress. Zinc stimulates the MTs, functioning as cytoprotective agents and electrophilic scavengers, thereby alleviating oxidative stress-induced cellular damage. More importantly, Zn facilitates the activation of GSH and catalase, thereby augmenting cellular defense systems [56,57,58,59].

WHO designates a dietary reference value for Zn of 6.7 to 15 mg per day. Exceeding the recommended daily consumption may lead to symptoms like anemia, neutropenia, and Zn-induced copper insufficiency. The European Food Safety Authority (EFSA) establishes an acceptable consumption level at 25 mg per day, but the FDA permits 40 mg per day. More importantly, Zn deficiency is significantly more prevalent than Zn toxicity, while vegetarians are especially vulnerable to it due to their diets, which typically contain fewer Zn-rich foods, such as meat, and higher phytate, which majorly diminishes Zn bioavailability [60].

For the last two decades, several in vitro studies have suggested that Zn enhances the production of A20 and PPAR-γ, zinc finger proteins (ZFPs) with anti-inflammatory properties, leading to reduced nuclear factor kappa B (NF-κB) activation and downregulation of its target genes, including tumor necrosis factor (TNF-α) and interleukin 1 (IL-1). Zinc ions may inhibit NF-κB activation by disrupting IκB kinase activity and blocking cyclic nucleotide phosphodiesterase, and subsequently providing anti-inflammatory properties. Excessive ROS production or compromised antioxidant systems result in oxidative stress, which contributes to chronic conditions such as aging, cardiovascular diseases (CVDs), and NDDs. In this context, Zn supplementation has demonstrated efficacy in decreasing oxidative stress indicators and inflammatory cytokines, as well as reducing the occurrence of infections in healthy persons. Zinc exerts antioxidative effects through multiple mechanisms: (i) competitive binding with redox metals such as iron and copper, (ii) binding to R-SH groups to safeguard biomolecules from oxidation, and (iii) enhancing the activity of enzymes, inducing MT expression, as well as inhibiting pro-oxidant enzyme activities [59,61,62]. Numerous research studies have investigated the role of Zn in inflammation and antioxidation. For instance, ZnO NPs are involved in typical anti-inflammatory properties, specifically reducing the activity of pro-inflammatory cytokines, inhibiting mast cell proliferation, and suppressing the production of COX-2 and iNOS. Zinc supplementation in adults has shown advantageous anti-inflammatory and antioxidative benefits [63,64,65]. Based on the above discussion, the multifaceted biological activities of Zn in facilitating anti-inflammatory and antioxidant properties are crucial in sustaining immune function and overall health.

## 4. Zinc Deficiency and Its Adverse Effects: Significance of Zinc Supplementation in Human Health

Zinc is a vital mineral with several physiological roles required for sustaining human health, which is essential for immunological function, metabolic activities, enzymatic reactions, and other biochemical pathways. Zinc is a cofactor for more than 300 enzymes, participating in essential cellular processes such as antioxidant mechanisms, apoptosis, signal transduction, and nucleic acid metabolism. Thus, properly maintained Zn homeostasis is vital for overall health, as nearly 20 distinct Zn transporters (ZnTs) regulate cellular Zn levels. Zinc deficiency or excess can lead to adverse health effects in sensitive tissues and organs. Meanwhile, adequate Zn intake provides numerous health benefits, primarily by supporting immune function and reducing oxidative stress and inflammation [66,67,68,69,70,71]. Therefore, Zn supplementation has been associated with several health advantages, including:

- Enhancing immune function to combat infections and diseases effectively, and thereby reducing respiratory tract infections (RTIs); cold, flu, sinusitis, pneumonia, and COVID-19.
- It is mainly supporting protein synthesis, DNA synthesis, carbohydrate metabolism, and cell proliferation, which are essential for tissue repair. Furthermore, potentiating CNS development and protecting cardiovascular health (CVH) [72,73,74,75,76,77].

Overall, Zn supplementation as a food supplement offers a range of health benefits by supporting vital physiological processes and promoting overall wellbeing.

Many health complications are linked to nutritional Zn deficiency and have since been identified, including chronic diarrhea, alopecia, brain dysfunction, impaired wound healing, chronic inflammation, and liver diseases, which collectively impact the immune system [78]. Noteworthy, Zn deficiency adversely impairs hemostasis, which is associated with numerous diseases: sickle cell anemia, GI disorders, alcoholism, burns, aging, and various cancers. More importantly, Zn deficiency leads to the most prevalent congenital disorder, i.e., acrodermatitis enteropathica (AE), which is caused by mutations in genes responsible for maintaining Zn homeostasis [74,79,80,81,82]. Major causes of Zn deficiency include diseases and genetic disorders that affect Zn absorption and increase intestinal Zn loss, as well as the consumption of foods low in Zn or containing unavailable forms of Zn.

Zinc supplementation has shown numerous benefits, and the treatment with a physiological dose of Zn has been associated with increased immune efficiency: a 50% reduction in Down syndrome infections and a decreased onset of infections in HIV patients have been reported. Significant recovery was noticed in patients with sickle cell disease and cancer. In addition, Zn supplementation is beneficial for patients with AE, and has also shown positive therapeutic responses in conditions such as leprosy, shigellosis, chronic hepatitis C, the common cold, and acute diarrhea in children. Supplementation with Zn can be preventive for atherosclerosis, myocardial infarction (MI), and ischemia. In individuals with sickle cell disease, Zn supplementation has been shown to enhance growth and secondary sexual characteristics while also normalizing plasma ammonia levels. Zinc supplementation helps rectify deficient delayed-type hypersensitivity (DTH) deficiencies and reduces infection and pain crises [83,84,85,86,87,88,89].

Research indicates that decreased Zn levels are correlated with reduced insulin sensitivity, compromised insulin secretion, and elevated inflammatory markers: TNF-α, IL-6, and C-reactive protein (CRP). Patients exhibiting low Zn concentrations in the blood also experienced chronic diabetic complications, inadequate glucose regulation, and diminished β-cell functionality. Meta-analyses regarding Zn supplementation have demonstrated beneficial effects on plasma lipid profiles, fasting glucose levels, and glycated hemoglobin (HbA1c) [90,91,92]. Nevertheless, a few studies suggest that Zn supplementation may improve glycemic control in diabetic retinopathy and help manage chronic liver disease; it may also ameliorate diabetic endothelial dysfunction, potentially via restoring the GTP cyclohydrolase-1 (GCH1) activity. Moreover, during pregnancy, Zn supplementation may lower the risk of adverse outcomes, including preterm birth [93,94].

### 4.1. Zinc Supplementation in Cardiovascular Diseases

The significance of Zn in CVH is garnering heightened interest due to its crucial influence on the preservation of CV function. Disrupted Zn homeostasis and sustained inflammation are prevalent characteristics in several CVDs. Zinc deficiency also leads to CVDs; supplementing with Zn is a feasible therapeutic approach for CVDs. Low quantities of Zn are sufficient for the functioning of the extracellular matrix (ECM) in cardiac tissue, which can fight against oxidative stress. Zinc is essential for preserving the functionality of cardiac tissue, and its insufficiency was reported in heart failure (HF), where it combats oxidative stress [95,96].

Furthermore, Zn supplementation can mitigate cardiac oxidative stress and decrease apoptosis in individuals following gastric bypass surgery and those with cardiomyopathy. This suggests that Zn exerts a protective influence on cardiac cells by mitigating oxidative stress and preventing cell death. From earlier studies, Zn supplementation in rats has successfully mitigated the cardiac remodeling after the completion of the MI [97,98].

During inflammation, Zn regulates NF-κB, which is an inflammatory modulator, and by inactivating NF-κB, Zn diminishes the countenance of cytokines, subsequently inhibiting the chronic inflammation in CVDs. In this context, Zn supplementation evidentially demonstrates benefits, including diminishing myocardial oxidative stress, inhibiting apoptosis, and mitigating cardiac remodeling following MI. From the above discussion, it is noteworthy that maintaining optimal Zn levels in CVH is crucial in dealing with CVDs.

### 4.2. Zinc Supplementation in Neurodegenerative Disorders and Aging Process

The Zn^2+^ ions are essential for many downstream apoptosis processes in somatic cells, which can trigger ND processes, potentially mediated by Zn^2+^ transfer from presynaptic vesicles to intracellular centers. Mitochondrial damage and oxidative stress are key indicators of Zn^2+^-induced neuronal necrosis, where free Zn^2+^ ions absorbed by mitochondria produce ROS, pro-apoptotic proteins, and mitochondrial dysfunction. The release of Zn^2+^ ions, either chemically induced or mediated by microglia, can stimulate 12-lipoxygenase, resulting in increased p38-mediated K^+^ efflux through newly formed Kv2.1 channels. Reactive oxygen species produced by microglia interact with reactive chemical elements to activate apoptosis through the apoptosis signal-regulating kinase 1/p38-mediated pathway and the Kv2.1-mediated K^+^ current surge. The Zn^2+^ ions are also necessary for p75 neurotrophin receptor-induced cell death, reducing K+/Cl- co-transporter 2 activity under oxygen-glucose deprivation conditions. The effect of Zn^2+^ exposure varies: short-term high Zn^2+^ concentrations cause necrosis and caspase activation, while prolonged low Zn^2+^ exposure triggers apoptotic and caspase-dependent pathways [99].

**Figure 4:**
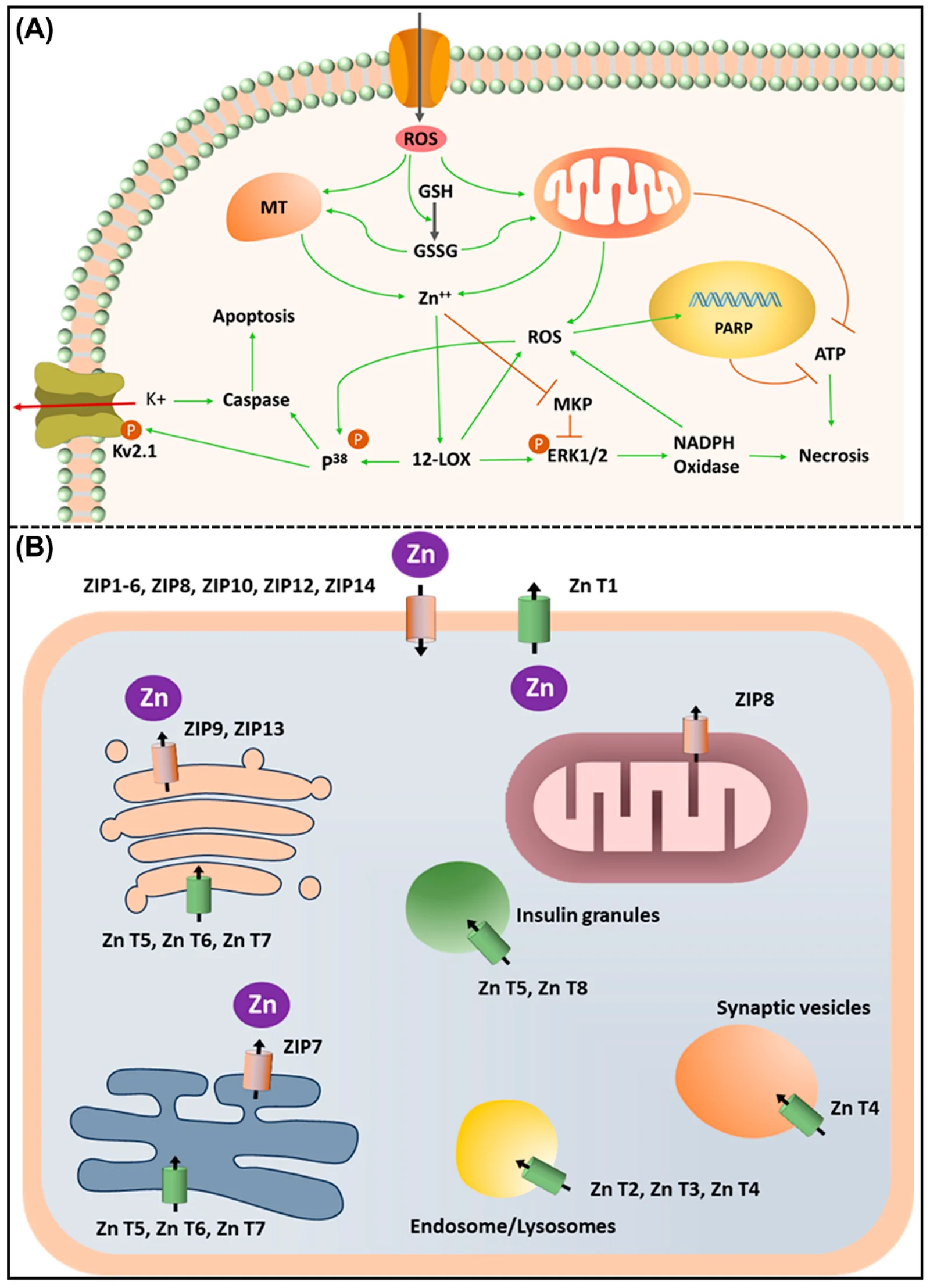
(**A**) Mechanism of neuronal cell death mediated by zinc: Zinc induces the production of ROS by activating enzymes such as NADPH oxidase and xanthine oxidase. The resulting ROS trigger mitochondrial dysfunction and activate apoptotic pathways, ultimately leading to neuronal cell death. (**B**) The 14-member ZIP family, which contains eight putative transmembrane domains, increases cytoplasmic Zn levels by facilitating the influx of Zn from either intracellular organelles or the extracellular environment.

Both NADPH oxidase (NOX) activation and neuronal death are linked to free zinc. The synaptically released Zn enters postsynaptic neuronal cells and regulates intracellular signaling. Neurotransmitter synaptic Zn may signal intracellularly, and Zn influx causes neuronal cell death in neurological conditions such as cerebral infarction, traumatic brain damage, hypoglycemia, and epilepsy. Zinc buildup in mitochondria and NOX activation are known to cause oxidative damage when cell Zn content rises fast. The poly ADP-ribose polymerase (PARP) is a crucial apoptotic marker and cell death regulator; the oxidative damage activates PARP, which depletes energy metabolites and kills cells. Due to severe damage, prolonged PARP activation depletes nicotinamide adenine dinucleotide (NAD) and adenosine triphosphate (ATP) levels, causing cell death. Significantly, Zn activates PARP; hence, higher intracellular Zn levels may boost PARP activity, and further, Zn overload increases intracellular ROS in an NADPH oxidase-dependent way, suggesting that ROS generation may contribute to zinc-induced PARP activation (Figure 4A) [70,100,101,102,103]. Zinc homeostasis is crucial for neuronal survival; on the contrary, its imbalance is associated with various NDDs like Alzheimer’s disease (AD), and in this context, Zn chelators have been serving as therapeutic agents. In vivo findings also suggest that metal chelators, i.e., clioquinol, can restore normal Zn homeostasis in somatic cells [81,104].

Due to nutritional and age-related physiological changes, older people often experience Zn insufficiency. Dietary changes can drastically lower Zn absorption and bioavailability. For example, meat, which is a significant source of Zn, is less consumed by older persons to lower cholesterol. However, they unfortunately consume more refined wheat products and fiber-rich meals with phytates, which can adversely limit Zn absorption. Zinc deficiency in older people is generally physiological, where certain conditions, such as depression, dental difficulties, and GI abnormalities, can significantly limit Zn intake. Clinically speaking, age-related changes in subcellular processes are crucial and impair Zn homeostasis by reducing its absorption and retention [105,106].

Zinc deficiency has detrimental effects on immunosenescence, which is an age-related decline in the immune function, and results in intense infections. In this regard, WBCs and NK cells need Zn as a cofactor for delivering an effective immune response. For instance, seniors who supplement with Zn can regulate MTs, which sequester Zn^2+^ ions intracellularly and deliver better immune and stress responses [107].

Zinc also protects against oxidative stress, a primary cause of aging and age-related disorders, which is a key component of SOD, and neutralizes ROS to protect cells. Zinc reduces the risk of older chronic illnesses such as CVDs, NDDs, and malignancies by inhibiting oxidative stress. Zinc supplementation can reduce the buildup of Cu^2+^ ions and its deleterious consequences in Wilson’s illness [69,83,84]. This therapy emphasizes Zn’s role in metal homeostasis and the prevention of cellular damage.

The participation of Zn in DNA repair pathways helps preserve genomic integrity and prevent age-related disease mutations. Normal Zn levels help repair DNA, thereby lowering the risk of cancer and other genetic disorders. Zinc is essential for healthy aging due to its functions in immunological function, antioxidant defense, DNA repair, and cellular homeostasis. The elderly must consume sufficient Zn through food or supplements to avoid Zn deficiency and can enhance immune response as well as improve health and lifespan, especially in aging populations.

## 5. Role of Zinc, Zinc Importers, and Transporters in the Immune System and Associated Disorders

The interplay between various genetic, immunological, and biochemical processes requires Zn as an effective supplement. A thorough focus on divergent molecular mechanisms driven by Zn will provide better insights about immunohomeostasis during pathological processes. The following sections discuss typical molecular mechanisms, which shed light on the Zn supplementation.

Zinc stabilizes process intermediates and aids substrate binding enzymatically. In tissue remodeling, Zn-dependent metalloproteases, such as matrix metalloproteinases (MMPs), degrade ECM proteins using Zn^2+^ ions and regulate transcription. Most DNA-binding proteins and transcription factors contain ZFPs, which are structural motifs stabilized by the binding of Zn^2+^ ions to cysteine and histidine residues. Primarily, ZFPs control gene expression and mediate cell development and differentiation, as well as support T cells and assist in adaptive immunity [80].

Zinc is essential for immune system function, regulating immunological response in numerous ways. This section discusses the role of Zn in immune cell growth and the production of cytokines and antibodies, as well as pathogen barrier integrity. Zinc guides immune cells to function appropriately and enables them to combat infections [108]. The tragic interplay between Zn deficiency and dysregulation of cytokines can interrupt immune homeostasis and precisely compromise host immune response. Proper Zn supplementation ideally delivers structural and functional integrity to the epithelial cells, which potentially fight against pathogens. Epithelial cells rely on Zn for proliferation, differentiation, and the maintenance of barrier function, thereby leading as the first line of defense against infections [79,80,85].

Zinc affects several cellular and molecular systems in the innate immune system that recognize and eliminate infections. Zinc is essential for the activation of neutrophils, macrophages, and dendritic cells. The Zn^2+^ ions activate immune cell signaling and phagocytosis, as well as the synthesis of antimicrobial peptides (AMPs), pro-inflammatory cytokines, and interferons (IFNs). Among these, specifically, IFNs limit viral replication and activate immune cells, enhancing antiviral immunity. Furthermore, epithelial cells contribute robust physical barriers to prevent pathogen invasion, and unfortunately, Zn deficiency inhibits epithelial barrier function, making infections more likely [86]. Therefore, Zn supplementation promotes cell proliferation, tight junction formation, and mucin synthesis to maintain epithelial barriers.

Moreover, Zn affects T and B cell formation, maturation, and function, affecting adaptive immune responses. In adaptive immunity, T cells coordinate pathogen-specific responses, and the thymus gland needs Zn to generate and mature T lymphocytes. Furthermore, Zn deficiency can alter T cell differentiation into pro-inflammatory or immunosuppressive phenotypes, affecting immunological homeostasis. Unfortunately, Zn deficiency inhibits T cell count and attenuates the immunological function. In this context, Zn supplementation preferably modulates cytokine production and signaling pathways to differentiate T helper (Th) cell subsets such as Th1, Th2, and Tregs. Moreover, B cells produce pathogen-neutralizing antibodies that are essential for humoral immunity, and in this regard, Zn is primarily needed for B cell activation, proliferation, and antibody synthesis, thereby potentiating immunological response [69,109,110]. Based on these superior roles of Zn supplementation in immune cell function, cytokine generation, antibody formation, epithelial barrier integrity, and antiviral activities, supplying appropriate Zn levels is crucial for immunological health and host defense against infections.

Both ZnTs and MTs function simultaneously for effective Zn homeostasis. Sufficient amounts of Zn are supplied to humans via consuming dietary foods, including fruits, nuts, red meat, oysters, whole-grain bread, and dairy products. Unfortunately, vegetables serve minimal portions of Zn to humans, which impedes Zn absorption [111].

The 14 members of the Zn- and iron-regulated ZIPs, comprising eight putative transmembrane domains, majorly coordinate Zn to enter into intracellular compartments and consequently enhance Zn levels in the cytoplasm (Figure 4B) [109,112]. Altogether, ZIPs, transporters, and MTs collectively regulate Zn homeostasis in cells, and the minor dysregulation among these pathways can lead to Zn deficiency disorders.

## 6. Dysregulation of ZIP Transporters in Cancer

Due to the vital role of Zn in DNA repair and immune system regulation, Zn deficiency may increase the risk of cancer. Transcription factors, such as p53, which orchestrates DNA damage repair responses, and antioxidant enzymes, like GPx and SOD, require Zn to function properly. The dietary Zn boosts the body’s antioxidant defense system, which fights oxidative stress and cancer. Zinc reduces oxidative stress-induced DNA damage and mutagenesis by mediating antioxidant enzymes that neutralize ROS. So far, many studies have dedicated their research to the dietary Zn deficiency and the cancer [113,114,115]. Zinc supplementation must be considered to maintain antioxidant status and DNA repair, which can resolve cancer.

The dysregulation of ZnTs is associated with various malignancies, including prostate, breast, lung, ovary, and pancreas. The abnormal expression of ZnTs, such as upregulation and downregulation, leads to cancer. More specifically, in prostate cancers, the upregulation of ZnTs causes increased Zn absorption into cancer cells, which enables Zn influx and triggers tumor-promoting signaling pathways and finally develops malignant tumors. For instance, the overexpression of ZnT (ZIP6) is attributed to the primary and metastasis of prostate glands [116]. The dysregulated Zn homeostasis impairs DNA repair, causing genomic instability and enabling cells more prone to cancer [117]. Addressing the dysregulation of ZnTs in cancer care is clinically relevant since epidemiological studies have linked abnormal function of the ZnTs to cancer progression.

Cancer treatment may modify Zn homeostasis and slow tumor growth by targeting dysregulated ZnTs. Therapeutic approaches that restore Zn homeostasis may benefit a variety of cancer types because ZnTs drive carcinogenic signaling pathways. Understanding the complex relationship between ZnTs and cancer etiology can lead to new cancer treatments and diagnostics. The entire therapeutic potential of targeting Zn homeostasis in cancer therapy requires further investigation into ZnT dysregulation mechanisms.

### 6.1. Role of ZIP1 in Prostate Cancer Development

The ZnTs, especially ZIP1, transport Zn^2+^ ions into prostate epithelial cells in normal prostates. This Zn influx inhibits mitochondrial aconitase (Aco2), a citric acid cycle enzyme, which is crucial to prostate function. Zinc hinders citrate oxidation by blocking Aco2, causing prostate epithelial cells to accumulate citrate. The citrate buildup lowers ATP synthesis, preventing cellular development under normal physiological circumstances. The overexpression of Ras-responsive element binding protein 1 (RREB1) leads to dysregulation of Zn influx in prostate cancer cells. The cancer cells have less Zn because RREB1 downregulates ZIP1 [118,119]. Thus, decreasing Zn levels in cancerous prostate cells increases ATP generation via reducing Aco2 inhibition. Increased ATP availability drives the energy needs of malignant cells, allowing for unrestrained multiplication and survival.

**Figure 5:**
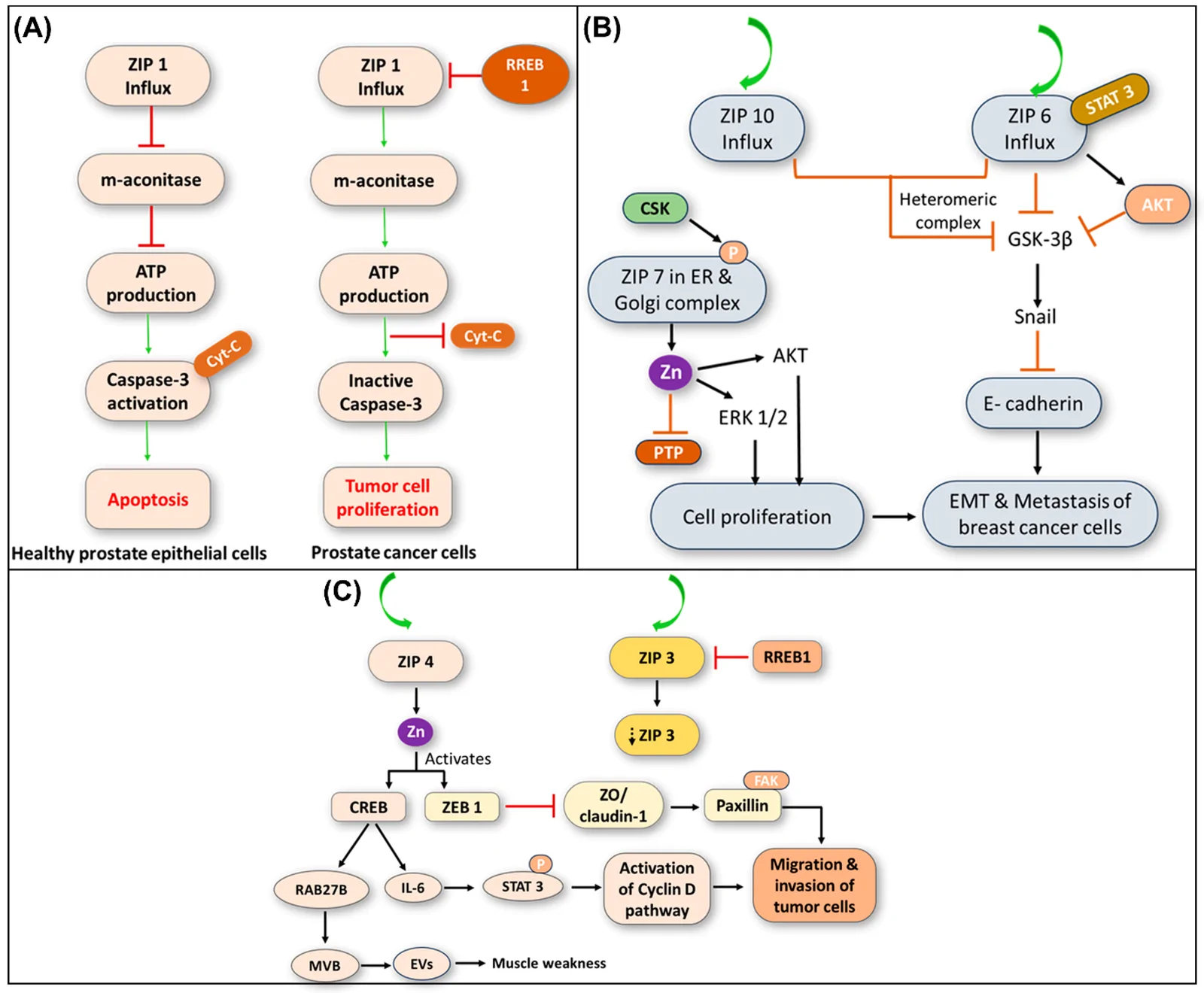
(**A**) The protective effect of Zn against the development of prostate cancer. In healthy prostate epithelial cells, the ZIP1 transporter plays a critical role in Zn uptake. In prostate cancer cells, ZIP1 expression is significantly reduced compared to normal prostate cells. This downregulation leads to decreased Zn uptake, which may contribute to prostate cancer growth and progression. (**B**) Overview of the functional roles of ZIP6, ZIP7, and ZIP10 in the molecular mechanisms of breast cancer. (**C**) Schematic illustration of ZIP4 overexpression in the promotion of pancreatic cancer.

The dysregulated Zn homeostasis in prostate cancer cells affects apoptotic pathways. Zinc releases cytochrome c from mitochondria and interacts with caspase-3, a mediator of apoptosis, in normal prostate epithelial cells [119]. The caspase-3 activation causes apoptosis, controlling cell proliferation and tissue homeostasis. However, prostate cancer cells with ZIP1 deficiency and low intracellular Zn levels produce cytochrome c and activate caspase-3 poorly. Thus, apoptotic pathways are repressed, allowing cancer cells to proliferate uncontrollably and advance malignancy. Notably, ZIP1 deficiency-mediated Zn homeostasis dysregulation in prostate cancer cells alters cellular energetics and apoptotic pathways, promoting tumor growth (Figure 5A). Understanding the complex molecular pathways of ZnT deregulation will help explain the development of prostate cancer and suggest future treatment options. Clinical scientists and medical professionals must understand these pathways to produce successful prostate cancer treatments.

### 6.2. Role of ZIP6, ZIP7, and ZIP10 in Breast Cancer Development

Normal mammary gland function, including lactation growth, requires Zn homeostasis. ZnT activity disruptions can cause Zn dysregulation and breast cancer. The ZIP6, a Zn importer protein, drives breast cancer growth. In luminal A breast cancer cells, ZIP6 expression is elevated and is phosphorylated by signal transducer and activator of transcription 3 (STAT3), a cancer-promoting transcription factor, upon activation [120]. The phosphorylated ZIP6 translocates to the plasma membrane, facilitating the entry of Zn into cancer cells.

The increased Zn influx via ZIP6 inhibits glycogen synthase kinase-3 beta (GSK-3β) and activates Snail, a ZFP implicated in epithelial–mesenchymal transition (EMT). The snail activation suppresses epithelial cadherin (E-cad), thereby preserving cell–cell adhesion and limiting tumor invasion and metastasis. Due to the disruption of the E-cad pathway, dysregulated ZIP6 activity increases cancer cell motility and metastasis. The ZIP10 is another ZnT linked to breast cancer development, and its high mRNA expression increases tumor invasiveness. Both ZIP10 and ZIP6 form a heteromer complex that enhances the inhibition of GSK-3β and E-cad inhibition, promoting tumor growth (Figure 5B). The deregulation of ZnTs leads to loss of cell adhesion, aiding in breast cancer invasion and metastasis. The tamoxifen-resistant (TamR) breast cancer cells depend on ZIP7, an ER, and Golgi ZnT. The CK2 phosphorylation of ZIP7 causes TamR cells to accumulate Zn. The phosphorylated Zn from the ER and Golgi apparatus stimulates downstream signaling pathways, including protein kinase B (AKT) and extracellular signal-regulated kinase (ERK 1/2), which encourages cell proliferation and invasion, as well as blocks tumor suppressor proteins like PTP, advancing breast cancer [116,121]. Overall, the deregulation of ZnTs such as ZIP6, ZIP7, and ZIP10 mediates cell progress, relocation, and incursion in breast cancer cells. Understanding the molecular causes of these dysregulations may provide new treatment strategies, and further investigation is needed to determine the functions of ZnTs in breast cancer etiology.

### 6.3. Role of ZIP4 in Pancreatic Cancer Development

The overexpression of ZIP4 in pancreatic cells may contribute to the onset and progression of pancreatic cancer. The ZIP4, a transmembrane protein, regulates Zn absorption and distribution in cells to maintain Zn homeostasis and stabilize the cellular function under physiological conditions. It is already widely known from earlier studies that ZIP4 is often overexpressed in pancreatic cancer, which greatly increases Zn absorption.

The overexpression of ZIP4 in pancreatic cancer cells activates signaling pathways for cell proliferation, survival, and migration.

- Excess Zn accumulation mediated by ZIP4 overexpression may also affect the tumor microenvironment, consequently leading to induced inflammation and suppressed immunity, and finally, facilitating a conducive milieu for substantial cancer cell proliferation.
- The overexpression of ZIP4 can trigger downstream events that further drive pancreatic cancer progression. For instance, the downregulation of ZIP3, another ZnT responsible for Zn influx, is inhibited by the RREB1, resulting in Zn loss and promoting metastatic progression [122,123].
- The ZIP4-mediated Zn influx activates ZF-transcription factors, facilitating the release of cancer-associated mutant genes from extracellular vesicles (EVs) through Ras-related protein Rab-27B (RAB27B)-mediated activation. Additionally, ZIP4 inhibits tight junction proteins such as zona occludens-1 (ZO-1) and claudin-1 via ZF E-box binding homeobox 1 (ZEB1), leading to increased tumor cell movement and motility through EMT (Figure 5C) [124,125].
- Targeting ZIP4 expression or its downstream signaling pathways presents a promising strategy for developing novel therapeutic interventions for pancreatic cancer. By disrupting Zn dysregulation mediated by ZIP4 overexpression, it may be possible to impede cancer progression and improve patient outcomes [126].

The dysregulated ZIP4 expression in pancreatic cancer promotes tumor growth and metastasis through abnormal Zn homeostasis. A deeper understanding of these pathways may help develop targeted therapeutics and provide insight into the pathophysiology of pancreatic cancer. The dysfunction of ZIP and ZnT transporters leads to several human disorders (Table 1 and Table 2). In this regard, Zn levels in serum and malignant tissues from cancer patients are valuable indicators for understanding the systemic Zn dysregulation (Table 3).

**Table 1:** Dysfunction of ZIP transporters (importers) and their Zn deficiency disorders.

ZIP Transporters (Importers)	Tissue Specificity	Functions	Mutation or Deficiency	Disorders	Ref
ZIP1	Adults and fetal tissues	Rapid uptake and accumulation of Zn in prostate cells	Single or double ablation of ZIP1	Prostate cancer; Abnormal embryonic development	[118]
ZIP2	Prostate and uterine epithelial cells	Uptake of Zn; Contact inhibition of normal epithelial cells	Single or double ablation of ZIP2	Abnormal embryonic development; Tumor genesis	[119]
ZIP3	Bone marrow, spleen, small intestine, and liver	Responsible for Zn influx transporter	Single or double ablation of ZIP3	Abnormal embryonic development	[122]
ZIP4	Kidney, small intestine, stomach, colon, jejunum and duodenum	Regulates Zn homeostasis	Loss of function and targeted disruption of the ZIP4 genes	AE, TEWL, ↑ IgE, and ↓ Th1/Th2 balance	[124]
ZIP5	Intestine, pancreas, liver, and kidney cells	Dietary uptake and homeostasis of Zn	Loss-of-function of ZIP5 gene	adHM	[125]
ZIP6	Prostate, placenta, and mammary glands	Act as a metalloproteinase	Loss-of-function of ZIP6 gene	Placenta cancer and metastasis	[121]
ZIP7	Mammary gland cells	Helps in Zn uptake	Loss-of-function of ZIP7 gene	Breast Cancer	[127]
ZIP8	Fibroblasts and chondrocytes	Causes Cd transport and toxicity in fibroblasts and chondrocytes	Hypomorphic mutation of ZIP8,Zn influx into cartilage chondrocytes lead to ↑ MMPs, Non-synonymous variant in ZIP8	Organ morphogenesis and hematopoiesis; Osteoarthritis due to ↑ IL-1β, IL-8 and TNF-α; ↑ MMPs and ROS; ↓ TH1 cytokines (IFN-γ, IL-2), and Schizophrenia	[128]
ZIP9	Human lymphocytes	Activation of AKT in response to BCR activation	Lack of ZIP9	↓ Fecundity; Egg viability; Retardations in the offspring growth	[129]
ZIP10	Mammary glands	Aid in the Zn influx	Lack of ZIP10	Impaired B-cell development; ↓ HIR	[121]
ZIP11	Testes, stomach, ileum, and cecum	For Zn transport	Lack of ZIP11	Zn deficiency	[129]
ZIP12	Brain and eye	For cellular Zn uptake	Targeted ZIP12 disruption	↓ PH in hypoxic conditions; Possible schizophrenia	[121]
ZIP13	Bone, teeth, and connective tissue	↑ Maturation: osteoblasts/chondrocytes/fibroblasts	ZIP13 deficiency	EDS; SCD-EDS	[129]
ZIP14	Mammalian cells	Cd transport and toxicity	ZIP14-deficiency homozygous loss-of-function mutations	↓ Growth, bone metabolism, and gluconeogenesis; Dystonia-parkinsonism; Neurodegeneration with hypermanganesemia in childhood.	[129]

Abbreviations: adHM: Autosomal dominant non-syndromic high myopia; AE: acrodermatitis enteropathica; EDS: Ehlers-Danlos syndrome; HIR: humoral immune response; MMP: matrix metalloproteinase; PH: pulmonary hypertension; SCD-EDS; Spondylocheirodysplastic Ehlers-Danlos syndrome; TEWL: trans epidermal water loss. “↑” means increase and “↓” means decrease.

**Table 2:** Dysfunction of ZnT transporters (exporters) and their Zn deficiency disorders.

ZnT Transporters (Exporters)	Tissue Specificity	Functions	Mutations	Disorders	Ref
ZnT1	Expressed in all tissues	Exporting metals from cytoplasm to extracellular medium	Targeted disruption of ZnT1	Embryonic lethality and abnormal vulva formation	[68]
ZnT2	Mammary gland, prostate, retina, pancreas, small intestine, and kidney	Accumulation of metals in organelles	Targeted disruption or mutation of ZnT2	Extremely low Zn content of breast milk	[70]
ZnT3	Brain, testes, and pancreas	Promotes the absorption of metal ions	Targeted ZnT3 disruption	Memory deficits with AD due to failure of Zn homeostasis proteins in neurons (MTIII- ZnT1-3)	[79]
ZnT4	Expressed in all cells		Loss-of-function mutation in ZnT4 (lethal milk mutant)	Post-natal lethality	[80]
ZnT5 and ZnT6	PM/Golgi/vesicular membranes	Accumulation of metals in vesicles for transportation	ZnT5 and 6 -deficiency	Severe osteopenia with impaired DTH	[82]
ZnT7	PM/Golgi/vesicular membranes	Accumulation of metals in vesicles, for transportation	ZnT7-deficiency	HFD-IGT	[86]
ZnT8	Pancreatic β cells	Maintain the concentration of blood glucose	ZnT8-deficiency	Impaired insulin secretion and crystal formation in DM	[87]
ZnT10	PM/Golgi/vesicular membranes	Accumulation of metals in vesicles, for transportation	ZnT10 mutation	Dystonia-parkinsonism with hyper manganesemia, polycythemia, and CLD	[87]

Abbreviations: CLD: chronic liver disease; DM: diabetes mellitus; DTH: delayed-type hypersensitivity; HFD: high fat diet; IGT: impaired glucose tolerance; PM: plasma membrane. “↑” means increase and “↓”means decrease.

**Table 3:** Deregulation of Zn levels in serum and tissues leading to various cancers.

Type of Caner	Serum Zn Levels	Tissue Zn Levels	Abnormal Transporters	Ref
Breast	↓	↑	ZIP6 ( ↑ ), ZIP7 ( ↑ ), ZIP9 ( ↑ ), ZIP10 ( ↑ ), ZnT2 ( ↑ )	[70,121,129]
Lung (NSCLC)	↓	↓	ZIP4 ( ↑ )	[124]
Nasopharynx (NPC)	↓	↑	ZIP4 ( ↑ )	[124]
ESCC	↓	↓	ZIP5 ( ↑ ), ZIP6 ( ↑ )	[121,125]
Ovarian cancer	↓	↓	ZIP4 ( ↑ )	[124]
Cervical cancer	↓	Uncertain	ZIP7 ( ↑ )	[121]
Prostate cancer	↓	↓	ZIP1 (↓) , ZIP2 (↓) , ZIP3 (↓) , ZIP4 (↓) , ZIP9 ( ↑ ), ZnT4 (↓)	[80,118,119,122,124,129]

Abbreviations: ESCC: esophageal squamous cell carcinoma; NPC: nasopharyngeal carcinoma; NSCLC: non-small cell lung cancer. “↑” means increase and “↓”means decrease.

## 7. Molecular Mechanisms, In Vitro, and In Vivo Studies

Divergent mechanisms such as competition for transporters and binding sites (DMT1, ZIP8/14), MT induction via MTF-1, Nrf2-driven antioxidant support, maintenance of epithelial barriers, and immune modulation are involved in mitigating HMs toxicity [127,128,129]. Yet, clinical translation requires in vivo evidence defining dose–response windows, timing, tissue distribution, and safety. Understanding the mechanistic biology of in vivo studies across HM exposures is crucial, which signifies practical biomarkers, such as plasma Zn, MT, urinary metals, and oxidative-stress indices, and guides evidence-based Zn supplementation in prevention and adjunct therapy [130,131,132].

**Figure 6:**
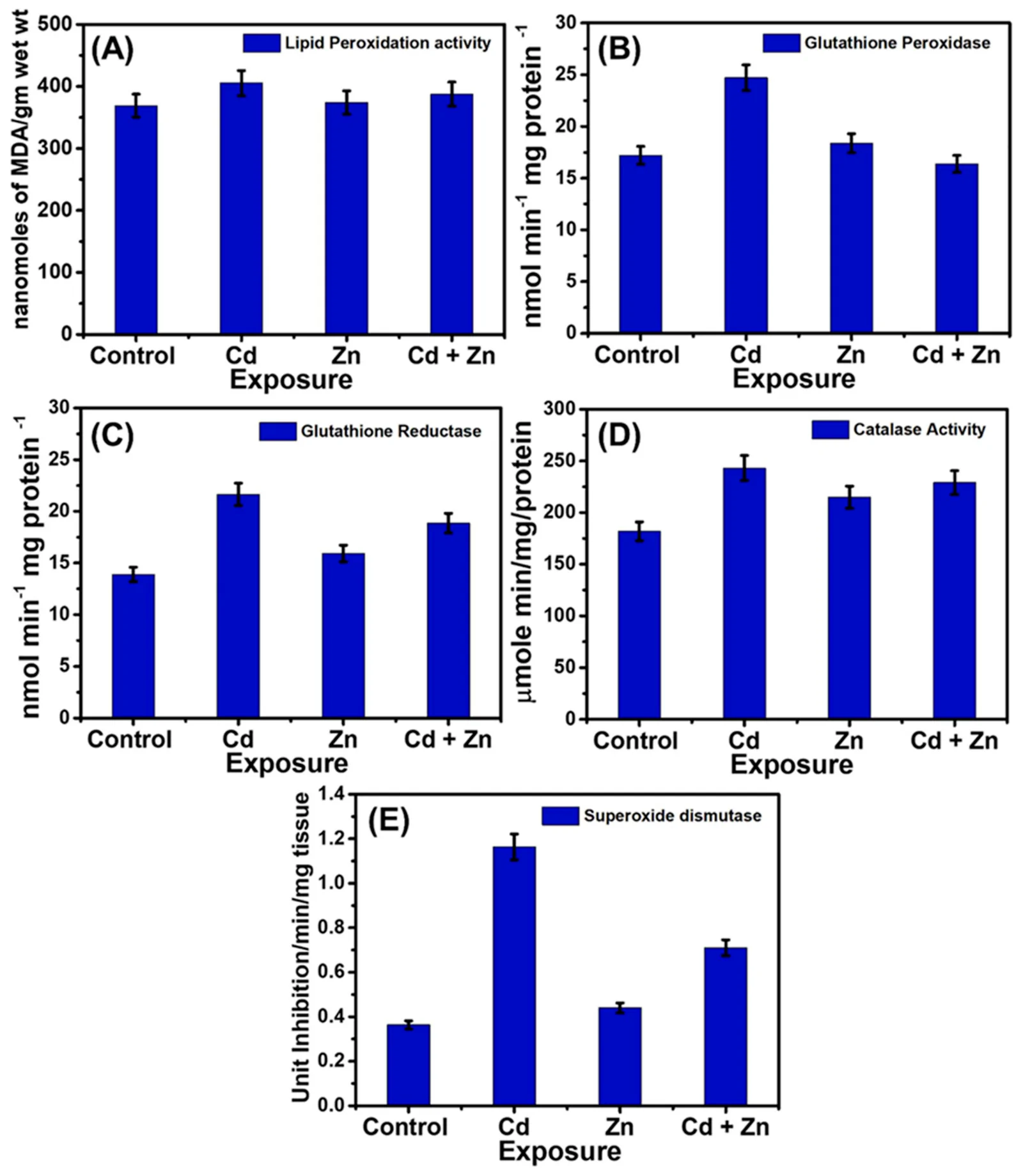
(**A**) Lipid peroxidase, (**B**) glutathione peroxidase, (**C**) glutathione reductase, (**D**) catalase, and (**E**) superoxide dismutase levels in liver tissue of zebrafish exposed to Cd and Zn (values represented are the mean of triplicates). One sample was made up of 14 fish livers (*n*  =  14 pools). Adapted from [133].

In zebrafish liver models of Cd injury, Zn consistently attenuates oxidative stress and restores antioxidant balance in hepatic tissue (Figure 6). Lipid peroxidation (LPO), measured by MDA, falls to near-control with Zn (≈387 nM MDA·g^−1^). Cadmium provokes compensatory rises in antioxidant enzymes—GPx to 24.0 nmol·min^−1^·mg^−1^ protein (control 17.5), GR to 23.5 nmol·min^−1^·mg^−1^ (control 14.3), catalase to 243.54 µmol·min^−1^·mg^−1^ (control 185.42), and SOD to 1.17 U·min^−1^·mg^−1^ (control 0.36). By contrast, Zn alone remains close to basal (e.g., GPx 18.2, GR ≈ control, catalase 210.31, SOD 0.45), and combined Cd + Zn partially normalizes these activities (GPx 6.0, GR 18.1, catalase 232.57, SOD 0.72), while returning LPO similar to control levels. Taken together, these patterns—Cd-induced LPO with enzyme upregulation and Zn-mediated re-equilibration—support a chemoprotective role for Zn (likely via thiol preservation, MT induction, and redox-gene modulation) that mitigates Cd-driven oxidative damage and helps restore homeostasis [133].

The As co-carcinogenesis is potentially harmful for human health; this molecular pathway is crucial for preventing and treating arsenic exposure. Zinc strongly induces MT, which binds toxic metalloids. Additional Zn may compete with arsenic for binding to GSH thiol groups at physiological pH, protecting against oxidation [134]. GSH and GSSG concentrations regulate the cell’s oxidative environment, which is normally lowered. The As-treated group had considerably lower liver GSH and total glutathione levels than the control group. The Zn and As supplementation increased GSH and total glutathione levels compared to As-treated rats. These mechanisms raise GSH and TG levels in liver tissue treated with As and Zn. In rats chronically exposed to As, Zn supplementation increases GSH levels in the livers [135,136]. The kidney GSH and TG levels did not differ significantly from the control in any treatment group. Additionally, exposure to As slightly lowered the levels of GSH. Previous investigations indicate that arsenic exposure alters the levels of GSH in kidney tissue [137,138].

**Figure 7:**
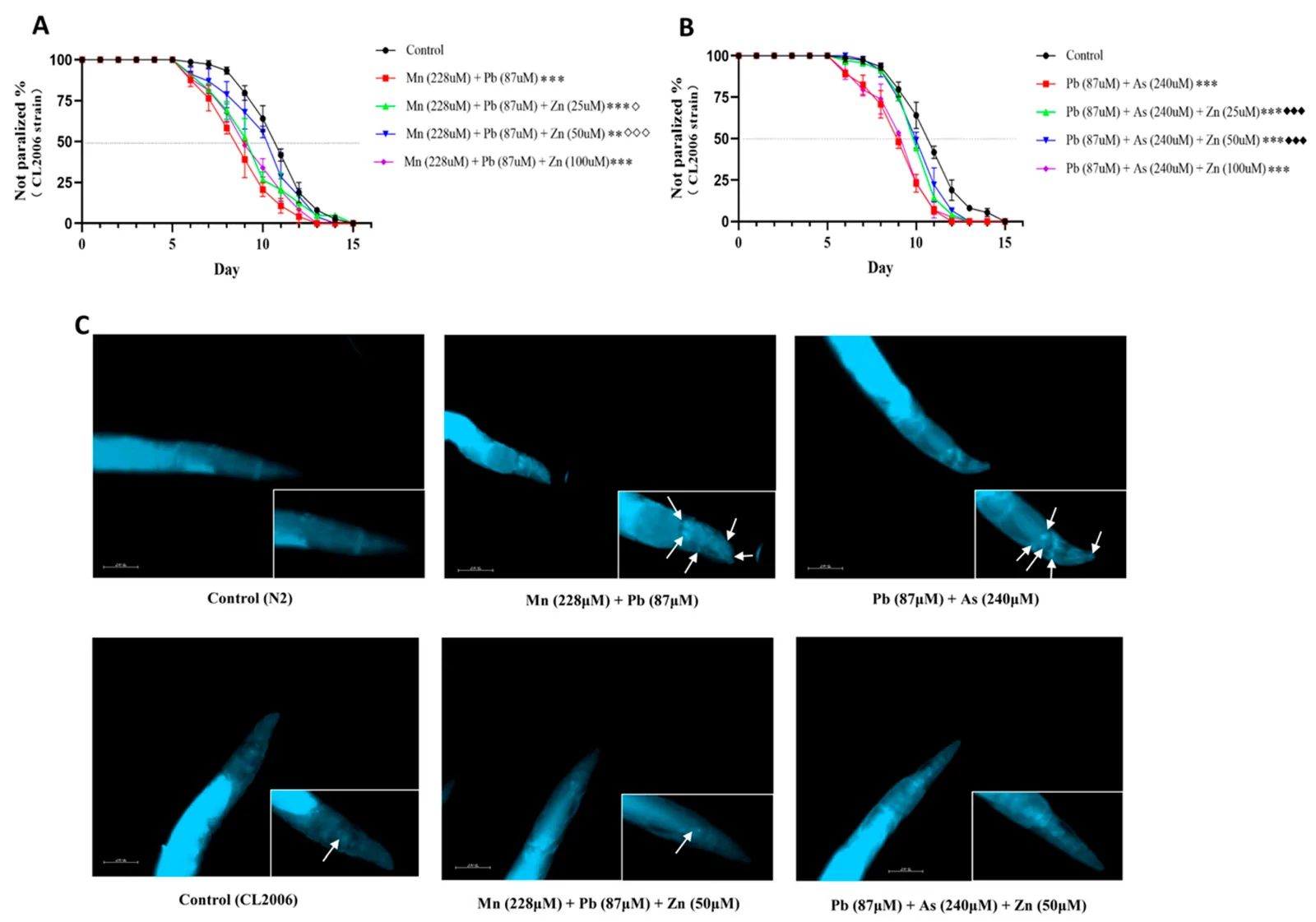
The effect of Zn homeostasis for paralysis behavior in transgenic nematodes CL2006. (**A**) The paralysis behavior of Mn + Pb mixture. (**B**) The paralysis behavior of Pb + As mixture. (**C**) Representative images of Aβ plaques in Mn + Pb and Pb + As mixtures. The Aβ plaques are located in the nematodes head region, indicated by arrows. Data are expressed as mean ± SD of three times independent experiments. The Kaplan–Meier statistics are indicated as follows: *, ◇, ◆: compared to control, Mn + Pb and Pb + As. Significant differences are denoted as ◇: *p* < 0.05, **: *p* < 0.01 and ***/◇◇◇/◆◆◆: *p* < 0.005. Adapted from [139].

Neurotoxicity can vastly be triggered by the formation of amyloid beta (Aβ) in different hierarchical organisms from nematodes to humans. Cai et al. investigated the therapeutic potential of Zn supplementation on the neurotoxic effects of an HMs/metalloids mixture (Mn, lead, and As) in a nematode model (*Caenorhabditis elegans*) [139]. The paralysis rates of Mn + Pb and Pb + As significantly increased from day 5 to day 15 compared to the control (*p* < 0.05). The paralysis rates of the *C. elegans* subjected to Mn + Pb and Pb + As were 50% on days 8 and 9, respectively, while the control group exhibited paralysis on day 11. Following the 50 μM Zn treatment, the 50% paralysis rates of the *C. elegans* subjected to the Mn + Pb and Pb + As combinations were prolonged until day 10 (*p* < 0.05) (Figure 7A,B). Furthermore, thioflavine S (ThS) staining was conducted on transgenic nematodes CL2006 to identify Aβ deposition, which demonstrated that metal combinations facilitated Aβ deposition, whereas Zn supplementation has diminished conveniently (Figure 7C). Thus, Zn homeostasis may impede Aβ aggregation triggered by multiple metal combinations.

**Figure 8:**
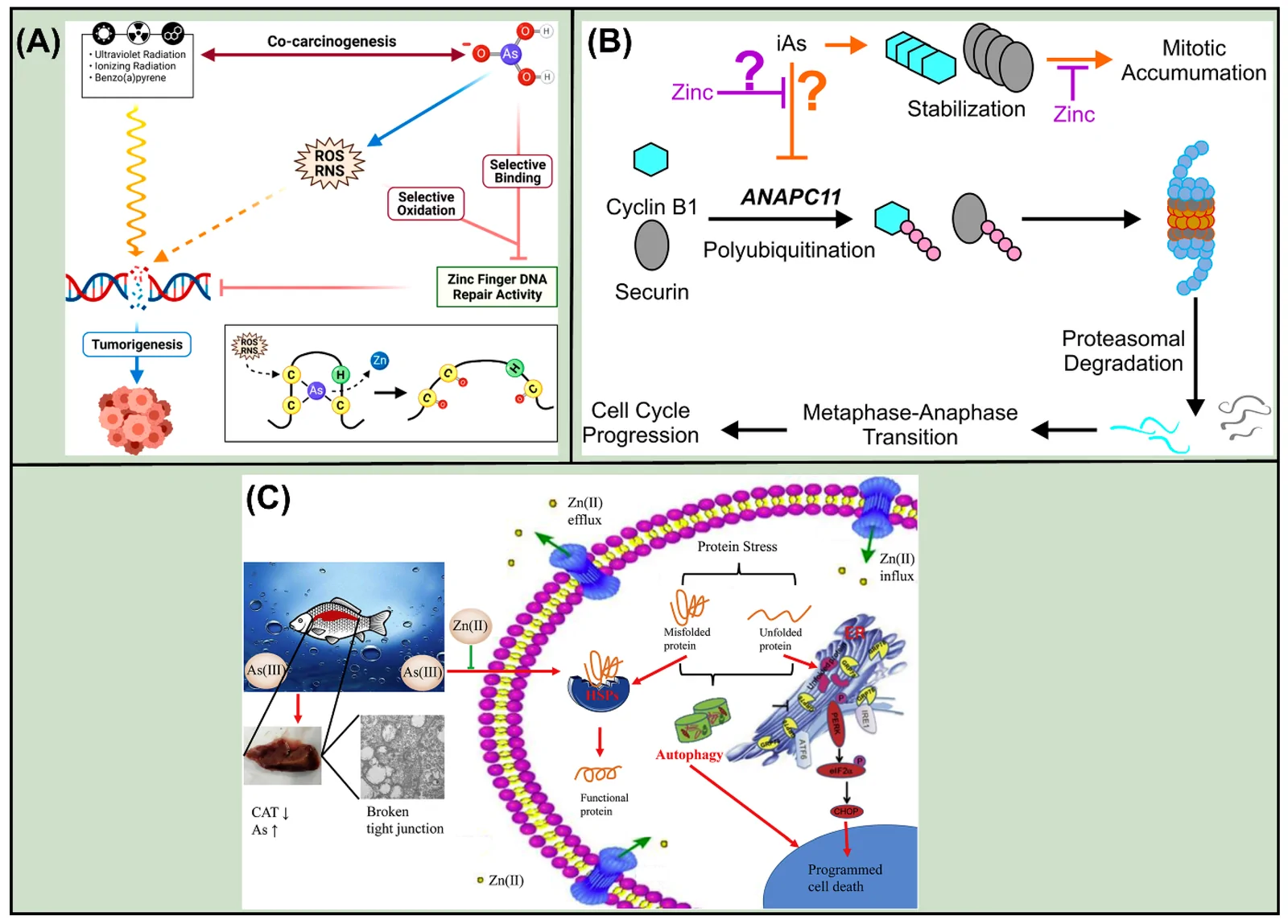
(**A**) Schematic illustration of arsenic co-carcinogenesis. Adapted from [140]. (**B**) Proposed mechanism of action by which iAs exposure induces mitotic accumulation. iAs exposure stabilizes cyclin B1 and securin preventing cells from progressing to anaphase from metaphase. Adapted from [141]. (**C**) Schematic depiction of the mechanism underlying zinc relieves arsenic-induced renal toxicity in common carps. Adapted from [117]. The arrows indicate the decreased catalase and increased arsenic.

Zhou and his research group suggested a molecular mechanism for selective arsenic binding to zinc finger proteins and the role of ROS. This mechanism shows how arsenic inhibits DNA repair and their interaction with Zn fingers, the predominant molecular mechanism for the co-carcinogenicity of eco- and pharmaceutical toxicants [140]. Banerjee et al. suggested that iAs exposure causes mitotic accumulation in human keratinocyte cells, probably via inhibiting cyclin B1 and securin breakdown [141]. Furthermore, Wang and his colleagues infer that As causes imbalance of the protein homeostasis in common carp kidneys, where Zn supplementation improves arsenic-induced kidney damage by modulating oxidative stress, HSR, ER stress, and autophagic process, as well as affecting the PI3K/Akt/mTOR pathway (Figure 8A–C) [117].

Respiratory disorders such as chronic obstructive pulmonary disease (COPD) are mainly caused by the exposure to cigarette smoke (CS), which constitutes the hazardous Cd in the concentration range of 1.56 to 1.96 μg. In smokers’ lungs, Zrt- and Irt-like protein 8 (ZIP8) transports Zn and Cd into cells substantially as compared to that in nonsmokers. In this scenario, it is crucial to understand whether diet or the ZnT, i.e., ZIP8, affects Zn homeostasis and enhances lung damage after extended CS exposure. The overexpression of ZIP8 increased lung Cd and tissue loss, while deletion of ZIP8 induced a Zn shortage and alveolar disintegration. In addition to dietary Zn intake, genetic polymorphic variation may perturb Zn and Cd transport into lung cells, which may be a risk factor for COPD in chronic smokers. Given the high Cd content in CS, dietary Zn consumption and genetic variation in Zn homeostasis proteins contribute to COPD development. Therefore, it is mandatory for understanding disease pathophysiology and compensating with sufficient micronutrient-based therapies that optimize risk-reduction efforts beyond smoking cessation [142].

**Figure 9:**
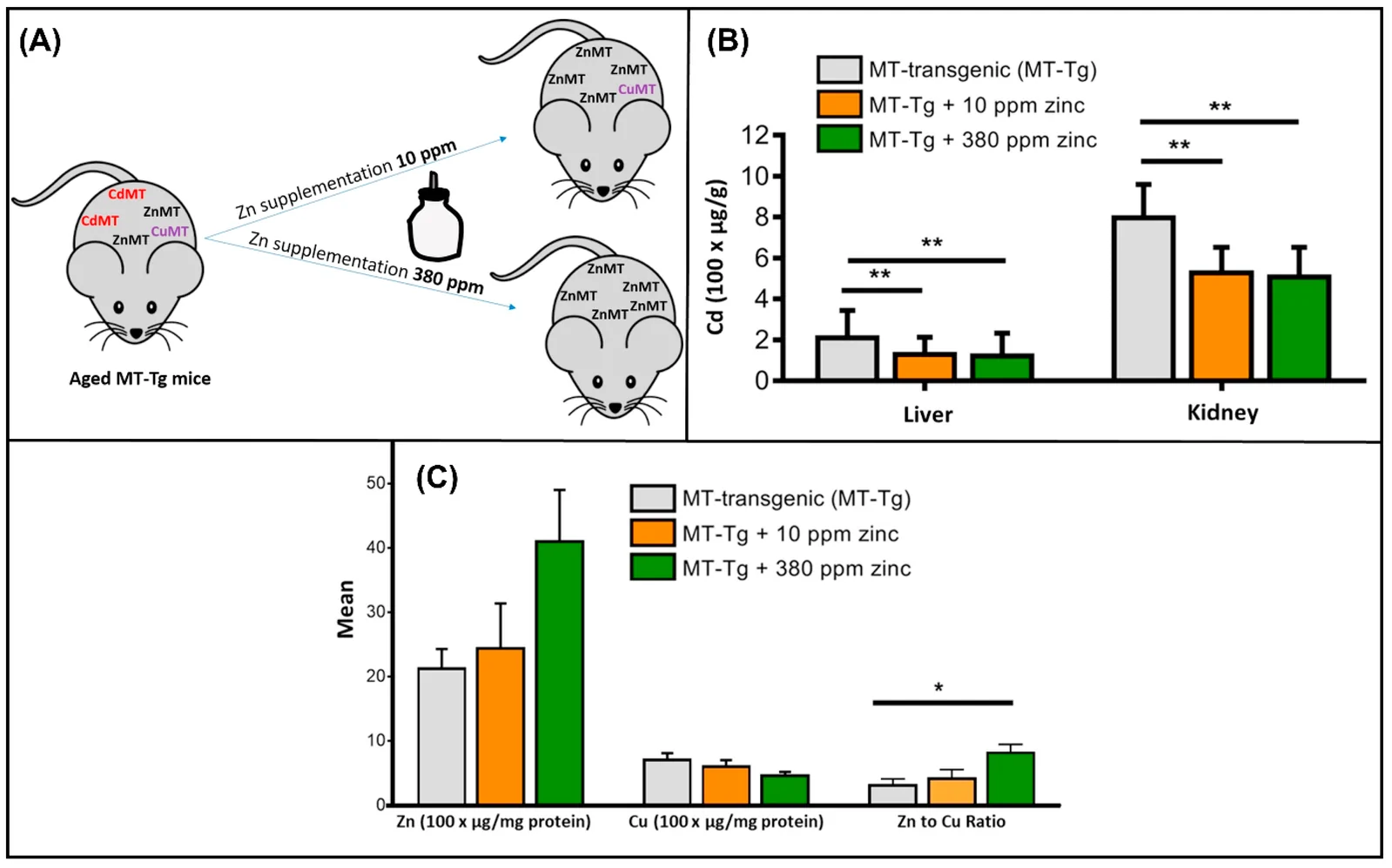
(**A**) Schematic representation for the Zn supplementation in aged MT1-tg mice. (**B**) Effect of Zn supplementation (1 month) on tissue levels of Cd, Zn and Cu in aged MT1-tg mice. A: Effect of Zn supplementation (1 month) on liver and kidney levels of Cd in aged MT1-tg mice (age ≥ 610 days, n = 4–6 per group). (**C**) Effect of Zn supplementation (1 month) on Cu and Zn levels measured in liver lysates of aged MT1-tg mice (age ≥ 610 days, n = 5 per group). Adapted from [143]. “*” and “**” indicate the statistical analysis.

As Zn deficiency is linked to higher Cd levels, effective Zn supplementation can mitigate compromised Cd levels. In a study, Pabis et al. observed a >30% decrease in kidney and liver Cd levels in Zn-supplemented old MT transgenic (MT1-tg) mice compared to controls. MT1-tg mice showed a comparable proportionate drop in both organs, despite a 5-fold greater Cd level in the kidney compared to the liver. Interestingly, this decrease was unaffected by Zn supplementation dosage, and Zn replaces Cd at ligand binding sites, presumably MT1. Alternatively, Zn may block Cd transport via DMT1 or Zrt/Irt-related protein (ZIP8), and the Cd displacement and decreased absorption happened in the initial phase of Zn supplementation. Authors compared Zn and Cu levels in livers from Zn-supplemented and control MT1-tg mice to determine if Zn supplementation affects Cd tissue concentrations and metal storage (Figure 9A,B). Specifically, the Zn levels in liver protein extract are very variable and increase with Zn supplementation, and the Zn-to-Cu ratio was considerably greater in mice treated with 380 ppm Zn compared to controls. Even a smaller Zn dosage can lower tissue Cd levels without impacting liver Cu or Zn levels, and the mice treated with 10 ppm Zn adapted to the new Zn-enriched environment and regained equilibrium (Figure 9C). The detected Zn levels show more variability than Cu and Cd levels, which are due to interindividual variabilities [143]. Significantly, the effective dropping of Cd accumulation by a low dose of Zn at high MT expression offers an excellent basis for future in vitro and clinical investigations on Zn supplementation’s effects on cellular Cd accumulation and Cd-related diseases.

In another study, Titus et al. evaluated tissue arsenic and Zn levels following oral arsenite, Zn, or arsenite + Zn administration. Six-week Zn supplementation had minimal effect on plasma Zn compared to controls. Arsenite exposure increased total tissue arsenic, with the kidney having the substantial amounts relative to the liver or spleen. Zinc supplementation has decreased total As in rats’ livers, kidneys, spleens, and lungs one month after exposure. In mice exposed to As-73 (As-73), Zn pretreatment reduced arsenic in the kidney and spleen 2 h later, and moreover, arsenite treatment of mice lowered plasma Zn levels without affecting magnesium, iron, or copper, suggesting Zn-As interactions are selective. Zinc supplementation effectively reduced arsenic in arsenite-exposed animals’ liver, spleen, and plasma [144]. From these observations, it is significantly suggested that compared to arsenite exposure alone, Zn supplementation reduced tissue arsenic levels in mice. Till now, several studies have been devoted to the nutritional benefits of Zn on As-exposed in vivo models [140,145,146,147,148,149,150,151].

**Figure 10:**
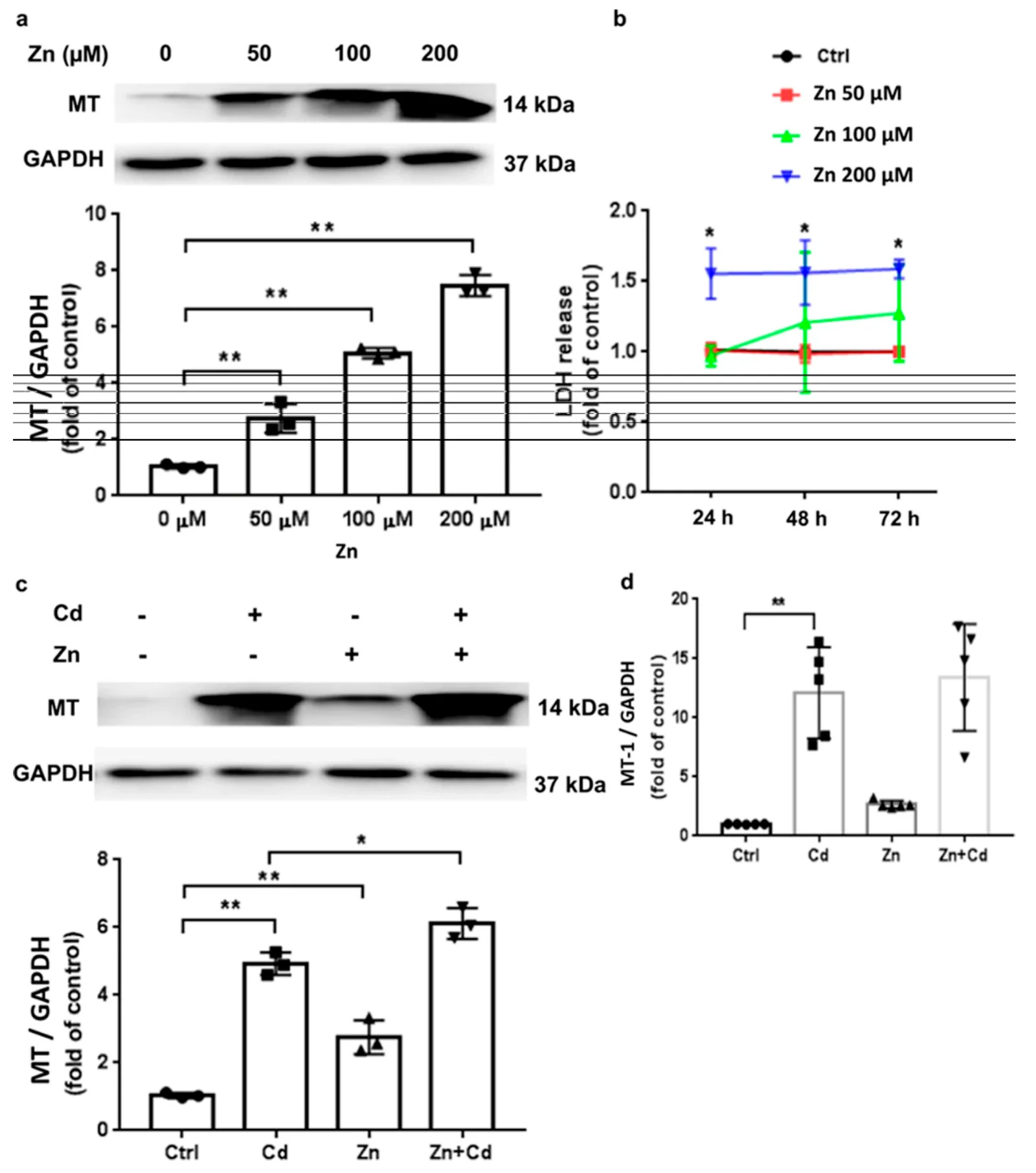
Zn and Cd effects on ECT MT expression. (**a**) MT expression after increasing Zn dose (50, 100, 200 µM) for 24 h. Group size is n  =  3 per dose. (**b**) LDH release after increasing Zn dose (50, 100, 200 µM) for different durations (24; 48; 72 h). Group size is n  =  3 per time point. (**c**) Effect of Zn (50 µM) and/or Cd (20 µM) on ECT MT protein content. Group size is n  =  3 per group. (**d**) Effect of Zn (50 µM) and/or Cd (20 µM) on MT-1 gene expression (qPCR). Group size is n  =  5 per group. * *p*<  0.05 and ** *p*  <  0.01 vs. corresponding Ctrl ECT. Adapted from [152].

**Figure 11:**
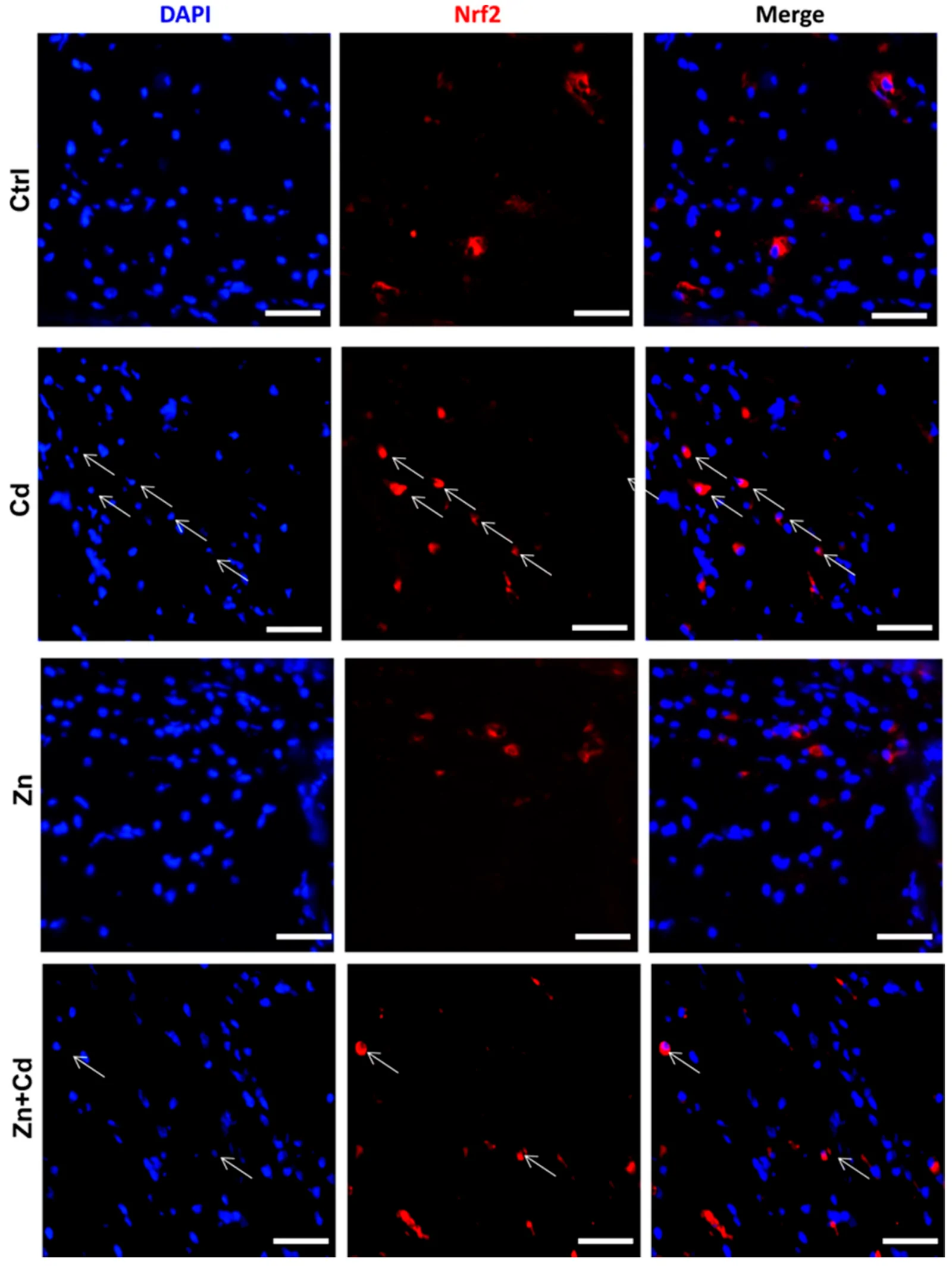
Evidence of ECT Nrf2 activation after Cd and/or Zn treatment. Representative ECT stained for Nrf2 (Red) and nuclei (DAPI, blue) following Zn (50 µM) and/or Cd (20 µM) for 24 h. Arrows indicate representative positive cells in which Nrf2 translocated into nucleus, ×40 magnification, scale bar  =  50 μm. Adapted from [152].

Recently, the biological effects of both Zn and Cd treatments on MT expression were investigated in mouse-engineered cardiac tissues (ECTs). Zinc caused a dose-dependent increase in MT expression and increased LDH release, indicating toxicity primarily at higher doses. However, after supplementing Zn (50 μM) in murine ECT, it has enhanced MT expression and substantial LDH release (Figure 10). The nuclear factor erythroid (Nrf2) nuclear translocation signaling is crucial for controlling oxidative stress in a myriad of diseases such as NDs, carcinogenesis, and HM toxicities. Evidently, after Zn treatment, MT-1 gene expression increased with MT protein expression, and specifically, the increased Nrf2 signaling process was moderately inhibited by Zn cotreatment (Figure 11) [152].

Further study suggests that administering Zn at the doses of 30 and 60 mg/L has increased element intake by 79% and 151%, respectively, preventing Cd buildup in brain tissue and preventing oxidative stress. A similar research group has demonstrated that increasing Zn administration by 79% and 151% did not affect the evaluated parameters in vascular tissue. Both Zn dose levels effectively mitigate the harmful effects of Cd on vascular tissue. Previous research indicates that supplementing with 30 mg Zn/L is more effective in reducing Cd toxicity in the liver than 60 mg Zn/L. Therefore, a 79% increase in Zn administration may be sufficient to defend target organs from the xenobiotic without causing negative outcomes. The percentage increase in Zn intake, which protects against Cd’s toxic effects on the CVS and other effects in male rats, is not significant, as the tolerable upper intake level for this element is 40 mg/day for men and women [153,154]. These in vivo studies suggest the feasibility of the Zn supplementation in mitigating HMs toxicity.

Noteworthy, Zn is a vital trace element for cell growth and tissue repair, which leads to it becoming an essential supplement for healing diversified wounds. Zinc deficiency leads to delayed wound healing in patients with chronic limb-threatening ischemia (CLTI), whereas oral Zn supplementation markedly enhanced healing rates (HR 2.30; *p* = 0.011) [155]. Moreover, in an aquatic model, i.e., common carp, a therapeutic dose of Zn sulfate (ZnSO_4_), equivalent to 1/5 of the predicted LC_50_ (2.09 mg/L), facilitated wound closure by exerting antioxidant activity and upregulating healing-related genes, including TGF-β1, COL1α1, VEGF-A, and FGF-7 [156]. In human clinical settings, daily supplementation with 50 mg of Zn in conjunction with ozone therapy accelerated the healing of diabetic foot ulcers via inhibiting inflammatory markers like serum C-reactive proteins [157]. Meanwhile, in vitro studies also demonstrate that therapeutic concentrations of Zn gluconate and Zn sulfate (ZnSO_4_) (0.1–0.001 µg/mL) significantly enhance the proliferation of fibroblasts and keratinocytes in nutrient-deficient conditions. Their antibacterial properties reduce the bacterial loads of *S. aureus* and *P. aeruginosa* by 4–5 log_10_, suggesting their therapeutic benefits in infected wounds [158,159]. Clinical evidence indicates that topical Zn formulations, such as ZnO pastes, accelerate the healing of chronic venous leg ulcers [160]. Polaprezinc, a Zn molecule, has shown dose-dependent correction of Zn deficiency, with 300 mg recommended for serum Zn levels below 70 µg/dL and 150 mg for elevated baseline levels [161]. Altogether, these studies indicate that Zn is effective in treating deficiencies and inducing wound healing via its immunomodulatory, antioxidant, and antibacterial potentials.

For decades, Zn has been claimed to be the crucial signaling molecule for regulating different biological processes in plants and animals. Zinc mitigates oxidative stress by eliminating free radicals through interactions with MTs, stabilizing proteins, and maintaining redox balance [162]. For example, when cells are exposed to Cd, MTs therapeutically activate Zn by trapping Cd and lowering the formation of ROS and protect cells from apoptosis [163]. It is already known that an excessive accumulation of Zn in mitochondria is able to disrupt complexes I and III of the electron transport chain (ETC), leading to the production of ROS, which exacerbates oxidative stress in cells [164,165]. This feature signifies the concentration-dependent effect of Zn. Furthermore, it shows some contradictory effects in both environmental and therapeutic contexts. In miners exposed to both Zn and Pb, lymphocytes manifested elevated ROS levels and mitochondrial damage, indicating that Zn exacerbates oxidative stress during HM co-exposure [166]. While Zn is necessary for the activation of microglial cells and normal immune function, high amounts can hinder these processes and generate free radicals, which could be harmful to the nervous system [167]. Conversely, both acute and chronic Zn exposure in photosynthetic organisms such as *Chlorella vulgaris* results in elevated protein concentrations and enhanced antioxidant enzyme activity, particularly SOD. Increasing Zn levels could induce ROS levels approximately six times, which caused protein levels to drop and antioxidant abilities to be disturbed, leading to worsening oxidative stress [168]. Numerous toxicological studies also revealed the implicatory roles of Zn in managing ROS. At normal levels, Zn can mitigate the HM toxicity, while an excess of it can implicate the antioxidant mechanism and cause the oxidative stress [169].

## 8. Conclusions and Future Perspectives

Zinc, a vital trace element, plays a crucial role in growth, immune function, and DNA synthesis. Zinc may also reduce HM toxicity in humans, which is important because HM exposure causes neurological impairment, renal malfunction, and cancer. Several mechanisms signify Zn as a potential chemoprotective supplement in combating HM toxicity, as follows: (i) Zinc competes with HMs for enzyme, protein, and cellular structure binding sites, limiting their absorption and accumulation in vital organs, including the brain, liver, and kidneys; (ii) zinc is highly triggered by MT, which sequesters and excretes HMs, specifically in mitigating Cd toxicity, which adversely affects kidneys; (iii) zinc protects cells against oxidative stress-induced HMs through enhancing enzymatic activity in the humans and thereby enabling a favorable antioxidant effect; and (iv) zinc can outcompete HMs for binding sites and increase MT activity against HM toxicity. Therefore, maintaining an adequate intake of Zn through food or supplements is crucial for mitigating HM toxicity in humans. Moreover, this review also discusses the molecular processes by which Zn impacts immune activities and the physiological roles of ZIPs and transporters ZnTs in maintaining Zn homeostasis and treating Zn deficiency illnesses. Research studies extensively explored the lack of ZIP1 in prostate cancer, the therapeutic roles of ZIP6, ZIP7, and ZIP10 in breast cancer, and the overexpression of ZIP4 in pancreatic malignancies. However, previously, few reports discussed typical molecular processes, in vitro, and in vivo investigations of Zn supplementation against HM toxicity.

In this review, molecular mechanisms by which Zn protects against HM toxicity were investigated, and further detailing these pathways may provide new treatment targets for various health complications. Zinc supplementation should be practically validated in clinical studies for the inhibition of toxicity by HMs. Despite short-term studies being conducted on the effects of Zn supplementation, effective long-term investigations might reveal therapeutic potential related to Zn supplementation and HM health outcomes. To raise awareness regarding Zn supplementation among scientific communities, several key aspects need to be considered. These include (i) public health campaigns should emphasize the importance of balanced meals for maintaining healthy Zn levels; (ii) properly educating people on Zn intake and heavy metal risks can help them make informed food choices; (iii) by considering age, sex, and health conditions, regulatory organizations should set safe Zn supplementation standards; (iv) public health requires monitoring of Zn intake and legislation to prevent over-supplementation; and (v) researchers and healthcare professionals should collaborate and implement comprehensive health prevention, detection, and management approaches to address the multifaceted issues of HM toxicity. Prioritizing these aspects can educate people regarding the nutritional and protective roles of Zn towards the mitigation of HM toxicity and nurture possible strategies to preserve human health.

## Data Availability

No new data were created or analyzed in this study. Data sharing is not applicable to this article.

## Abbreviations

ZFP	Zinc Finger Proteins
ZIP	Zrt- and Irt-like protein
ZnT	Zinc Transporter
ZEB1	Zinc finger E-box binding homeobox 1
MT	Metallothionein
ECM	Extracellular matrix
NK cells	Natural killer cells
TNF	Tumor Necrosis Factor
GPx	Glutathione peroxidase
ILs	Interleukins
IFNs	Interferons
NOX	NADPH Oxidase
PARP	Poly ADP-ribose polymerase
ROS	Reactive oxygen species
RREB1	Ras-responsive element binding protein 1
RAB27B	Ras-related protein Rab-27B
Aco2	Mitochondrial aconitase
SOD	Superoxide Dismutase
MMPs	Matrix metalloproteinases
AMPs	Antimicrobial peptides
ATP	Adenosine triphosphate
NAD	Nicotinamide Adenine Dinucleotide
E-cad	Epithelial cadherin
GSK-3β	Glycogen Synthase Kinase-3 beta
STAT3	Signal Transducer and Activator of Transcription 3
